# Immunogenicity of pneumococcal vaccination in HIV infected individuals: A systematic review and meta-analysis

**DOI:** 10.1016/j.eclinm.2020.100576

**Published:** 2020-11-23

**Authors:** Hannah M.Garcia Garrido, Jenny L. Schnyder, Michael W.T. Tanck, Albert Vollaard, René Spijker, Martin P. Grobusch, Abraham Goorhuis

**Affiliations:** a Centre of Tropical Medicine and Travel Medicine, Department of Infectious Diseases, Amsterdam Infection & Immunity, Amsterdam Public Health, Amsterdam University Medical Centres (AUMC), Meibergdreef 9, Amsterdam, AZ 1105, The Netherlands; b Amsterdam UMC, University of Amsterdam, Department of Clinical Epidemiology, Biostatistics and Bioinformatics, University of Amsterdam, Meibergdreef 9, Amsterdam, The Netherlands; c Center for Infectious Disease Control Netherlands, National Institute for Public Health and the Environment, Antonie van Leeuwenhoeklaan 9, Bilthoven, The Netherlands; d Academic Medical Centre, University of Amsterdam, Medical Library, Amsterdam Public Health, Amsterdam, The Netherlands; e Cochrane Netherlands, Julius Center for Health Sciences and Primary Care, University Medical Center Utrecht, Utrecht University, The Netherlands

**Keywords:** HIV, Vaccination, Pneumococcal, Invasive pneumococcal disease, Pneumonia, 13valent, Conjugate vaccine, 23-valent, Polysaccharide vaccine

## Abstract

**Background:**

The objective of this systematic review and meta-analysis was to summarise the literature regarding the immunogenicity of pneumococcal conjugate vaccines (PCV) and pneumococcal polysaccharide vaccines (PPSV) in adult people living with HIV (PLWH) in the era of advanced combination antiretroviral therapy (cART).

**Methods:**

The systematic review protocol was published online (PROSPERO ID: CRD 42020153137). We searched Medline (Ovid), EMBASE (Ovid), and the Global Health Library for publications from 2000 to June 11, 2020. We included all studies in adult PLWH that reported vaccine immunogenicity outcomes. The primary outcome was seroconversion rate (SCR) after PCV, PPSV and PCV/PPSV combined. For random-effects meta-analysis, we included studies defining SCR as *a* ≥ 2-fold increase in IgG from baseline, and reporting SCR for serotypes 6B, 14, or overall SCR, 1–3 months after vaccination.

**Findings:**

Our search identified 1597 unique studies, of which 115 were eligible for full-text assessment. Of these, 39 met the inclusion criteria (11 RCTs; 28 cohort studies). A high degree of heterogeneity was observed. Nineteen studies were included in the meta-analysis. Pooled overall SCRs were 42% (95% CI 30–56%), 44% (95% CI 33–55%) and 57% (95% CI 50–63%) for PLWH who received PPSV, PCV or a combination of PCV/PPSV, respectively. Compared to PPSV alone, a combination of PCV/PPSV yielded higher SCRs (OR 2.24 95% CI 1.41- 3.58), whereas we did not observe a significant difference in SCR between PCV and PPSV23 alone. There were no statistically significant differences in geometric mean post-vaccination antibody concentrations between vaccination schedules. Vaccination at higher CD4 cell counts improved immunogenicity in 8/21 studies, especially when PCV was administered. No studies assessed the long-term immunogenicity of PCV followed by PPSV23. Quality of evidence ranged from poor (*n* = 19) to good quality (*n* = 7). A limited number of pneumococcal serotypes was assessed in the majority of studies.

**Interpretation:**

We show that the recommended immunisation schedule consisting of a combination of PCV13/PPSV23, is immunogenic in PLWH in the era of advanced cART. However, the durability of this vaccination schedule remains unknown and must be addressed in future research. Vaccination with PCV should be delayed until immunological recovery (CD4>200) in recently diagnosed PLWH for optimal immunogenicity. The evidence gathered here supports wide implementation of the combination of PCV/PPSV23 for all PLWH. We recommend reassessment of this strategy once higher-valent PCVs become available.

**Funding:**

HMGG is funded by a public research grant of ZonMw (project number 522004005).

**Research in context**

**Evidence before this study**

People living with HIV (PLWH) are at increased risk of pneumococcal disease. Although combination antiretroviral therapy (cART) substantially reduced the risk of opportunistic infections, the incidence of invasive pneumococcal disease and pneumonia remains five times higher as compared to the general population. Currently, a schedule combining PCV and PPSV is recommended for PLWH in most countries. In the past decades, many immunogenicity studies in PLWH have been published, using a number of different vaccination regimens. A large amount of these studies was conducted before cART became standard of care, and may therefore not reflect the current clinical situation of PLWH.

**Added value of this study**

Due to the absence of representative pneumococcal vaccine efficacy studies, immunogenicity studies remain the cornerstone of vaccination recommendations. To our knowledge, no published systematic review or meta-analysis on the immunogenicity of PCV and PPSV in adult PLWH exists. By only including studies published in the era of advanced cART (after 2000), we present results that best reflect the current situation for PLWH.

**Implications of all the available evidence**

The current literature supports the use of the combined vaccination schedule of PCV13 followed by PPSV23 for PLWH. This vaccination schedule yields the best overall immunogenicity in the era of advanced cART, with the majority of patients responding to vaccination. However, long-term immunogenicity of this vaccination schedule was not addressed in existing studies. Therefore, recommendations on the optimal timing of a booster dose cannot be substantiated. In addition, future studies should focus on long-term immunogenicity of the combined PCV/PPSV23 schedule in PLWH, as well as the immunogenicity of the promising 20-valent conjugate vaccine in PLWH.

Alt-text: Unlabelled box

## 1 Introduction

People living with HIV (PLWH) are at increased risk of pneumococcal disease, even in the era of advanced combination antiretroviral therapy (cART). A recent study showed an incidence rate of pneumococcal disease in PLWH of 190 per 100,000 patient-years of follow-up, compared to 38 per 100,000 in the general population [1]. Pneumococcal disease in PLWH often requires hospitalisation, and mortality rates range up to 25% [2]. In addition, the recurrence rate is high [3]. Therefore, pneumococcal disease remains an important problem in PLWH, contributing significantly to disease burden and health care costs [4].

To date, two types of pneumococcal vaccines are available: the T cell-dependent 13-valent conjugated pneumococcal vaccine (PCV13), and the T cell-independent 23-valent polysaccharide vaccine (PPSV23), with divergent immunological effects [5].

Most international guidelines recommend pneumococcal vaccination in PLWH with PCV13, followed 8 weeks later by PPSV23 [6,7]. However, the clinical efficacy of this combined vaccination schedule in PLWH is unknown. Therefore, HIV-care providers tend to be restrictive regarding vaccination, which is reflected by a low overall pneumococcal vaccine uptake amongst PLWH in many countries [8,9]. Studies on clinical vaccine efficacy require large numbers of participants. For example, the largest published study on clinical efficacy of PCV13 against pneumococcal disease among adults 65 years of age or older, included almost 85,000 participants [10]. Such numbers will likely never be reached in specific populations of increased risk, such as PLWH, or otherwise immunocompromised individuals. It is therefore not realistic to demand scientific proof on the level of clinical efficacy studies before accepting that vaccination of risk groups could be beneficial. The next best way of going forward is to look at immunogenicity, as surrogate marker for clinical efficacy [11]. That is to say, without a sufficient immunological response to (pneumococcal) vaccination, clinical protection against disease is unlikely. ^12^Several studies have addressed the immunogenicity of pneumococcal vaccination in PLWH, showing variable vaccination responses, depending highly on the type of vaccine used and immunological status of the studied population [5,^13^,14].

The objective of this systematic review and meta-analysis was to summarise the literature regarding the immunogenicity of pneumococcal vaccination in adult PLWH, in the era of advanced cART (since 2000) [15].

## 2 Methods

We adhered to the PRISMA guidelines for reporting of systematic reviews and meta-analysis [16]. The protocol was registered online in the PROSPERO systematic protocol registry (www.crd.york.ac.uk/prospero) ID: CRD 42020153137.

### 2.1 Search strategy and selection criteria

On November 27, 2019 we searched Medline (Ovid), EMBASE (Ovid), and the Global Health Library for studies evaluating *pneumococcal vaccination* in *patients with HIV*, with a last update performed on June 11, 2020. In addition, the International Clinical Trials Registry Platform (ICTRP) Search Portal and ClinicalTrials.gov were searched for ongoing, or completed unpublished trials. The conference abstracts segment in EMBASE (Ovid) was used to identify relevant conference abstracts. Authors of registered unpublished trials and conference abstracts were contacted to include their data in our systematic review. The full search strategy can be found in Supplementary File 1. Reference lists from selected studies and review articles were checked to identify any other eligible studies.

After removal of duplicates, two authors (HMGG and AG) independently screened titles and abstracts to select articles meeting the eligibility criteria (Box 2), using Rayyan software [17]. HMGG and AG assessed the remaining full-text articles. Discrepancies were resolved by discussion. If HMGG and AG did not agree after discussion, a third author (MPG) was consulted. If there were multiple reports of one study, only one was included.

The following study characteristics and outcome data were independently collected by HMGG (100%) and JLS (60%), and checked by AG (40%), with a standardised data extraction sheet: author name, country of study, publication year, study design, number of PLWH and HIV-negative controls, age, % of individuals on cART (defined as the use of at least 3 antiretroviral drugs), % of individuals with an undetectable viral load (threshold of original study was used), mean/median CD4 count, vaccination regimen, definition of SCR used in the study, type of serologic test used, serotypes assessed, interval between vaccination and antibody measurement, SCR after vaccination (6B, 14, overall), baseline and post vaccination GMCs (serotype 6B, 14), factors associated with non-response. In case of missing data, the authors of the original studies were contacted and given a deadline to provide the missing data. For inclusion in the meta-analyses, authors were contacted and asked to provide their data according to the definitions provided in Box 2.

HMGG and JS assessed the risk of bias in selected studies. Disagreement was solved by discussion. To assess risk of bias in cohort studies, we used a modified version of the Newcastle-Ottawa assessment tool for cohort studies [18]. In this tool, a maximum of nine stars could be awarded for eight different items in three domains: selection, comparability and outcome assessment. We converted the Newcastle Ottawa to the standards of the Agency for Healthcare Research and Quality - AHRQ (Supplementary file 2) [19]. To assess the risk of bias in randomised trials, we used the Cochrane Collaboration's tool [20]. We considered an RCT of good quality if all criteria were met (i.e. low risk of bias for each domain), or if one criterion was unclear (i.e. risk of bias not ascertainable); of fair quality if one criterion was not met (i.e. high risk of bias for one domain), or if two criteria were unclear; and of poor quality if two criteria were not met, or if one criterion was not met and one or more critereria were unclear.

### 2.2 Data analysis

The primary outcome of the systematic review was the SCR 1–3 months after vaccination with PCV,

PPSV23 or a combination of PCV/PPSV23, in adult PLWH. Secondary outcomes were: long term SCR (>2 years) after vaccination with PCV, PPSV23 or a combination o PVC/PPSV23; the strength of the immune response measured by GMC, IgG, or OPA-titres; the influence of CD4 count, cART use, and viral suppression on vaccine immunogenicity.

The primary outcome of the meta-analysis was the pooled SCR for serotypes 6B, 14, and the overall SCR (50–70% of measured serotypes) [21] after PCV, PPSV23, or a combination of PCV/PPSV23, in adult PLWH. We focussed on SCR for serotype 6B and 14 and overall SCR, as these outcomes were reported in sufficient studies to perform a meta-analysis, whilst the other serotypes were investigated in too few studies to meta-analyse. Secondary outcomes of the meta-analysis were: differences in SCRs, and in log-transformed GMCs, between different vaccination schedules, either including PCV only, or PPSV23 only, or a combination of PCV/PPSV23.

To address reporting bias, we checked if the study protocols of included studies had been previously published and if there had been any deviations**.** Authors of individual studies were contacted for additional data if specific data were not available in the manuscript. To address publication bias, we also included conference abstracts and completed unpublished trials.

### 2.3 Role of funding sources

HMGG is funded by a research grant of ZonMw (project number 522004005). The funder of the study had no role in study design, data collection, data analysis, data interpretation, or writing of the report. The corresponding author had full access to all the data in the study and had final responsibility for the decision to submit for publication.

## 3 Results

The initial search yielded 2037 records, of which 1597 remained after removing duplicates.

**Fig. 1:**
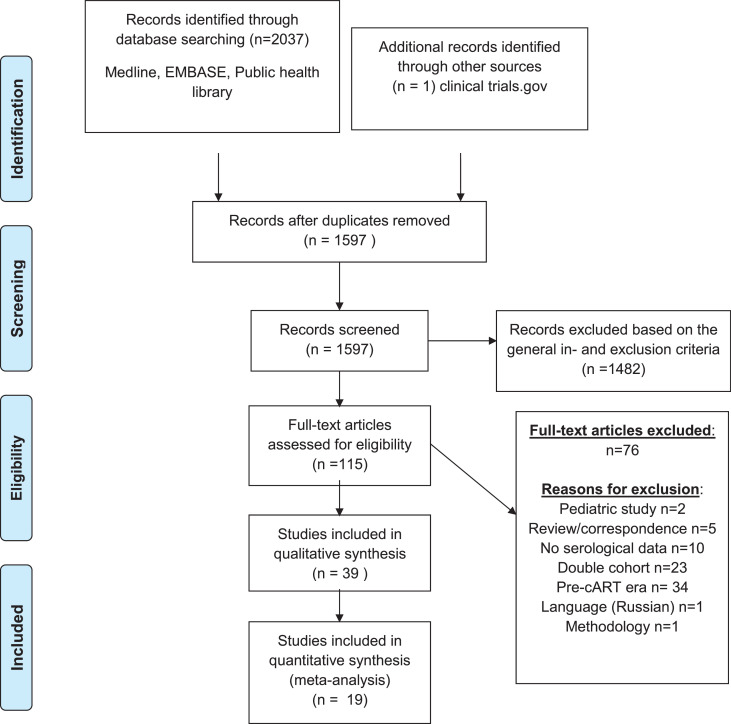
Flow-chart showing study selection process.

After screening titles and abstracts, 115 articles were assessed full text for eligibility, of which 38 met the eligibility criteria for this systematic review, and 19 for the meta-analysis. One additional recently completed unpublished clinical trial was identified from trial registries (NCT02717494), resulting in 39 included studies in total (Fig. 1).

We identified four ongoing or recently completed unpublished trials of interest, which did not have data available yet. First, NCT03480802, a recently completed phase 3 trial with 302 participants evaluated a 15-valent pneumococcal conjugated vaccine (V114, Merck Sharp & Dohme Corp.), followed by PPSV23 in PLWH. Second, NCT03039491, an RCT in the final recruitment stage, compared PCV13 to PPSV23 in elderly PLWH. Last, two non-randomised clinical trials (NCT02012309 and NTR7385) investigated pneumococcal vaccine responses to the recommended PCV13 followed by PPSV23 (*n* = 60 and *n* = 100 PLWH, respectively).

### 3.1 Study characteristics

The included studies comprised a total of 4649 PLWH and 250 HIV-negative controls. In total, 2437 individual PLWH received PPSV23, 1275 received PCV, 792 received a combination of PPSV23 and PCV, and 145 received a placebo. Most studies (27/39) were cohort studies [5,^13^.[22], [23], [24], [25], [26], [27], [28], [29], [30], [31], [32], [33], [34], [35], [36], [37], [38], [39], [40], [41], [42], [43], [44], [45], [46], 11/39 studies were RCTs [14,[47], [48], [49], [50], [51], [52], [53], [54], [55], [56], and one study was a combination a cohort study and an RCT [57]. Only 8/39 studies included a HIV negative reference group [27,^29^,^31^,^34^,[38], [39], [40],48]. Most studies (36/39) reported short-term immunogenicity (one month to one year after vaccination). Three studies were follow-up studies of previous trials and reported long-term immunogenicity [24,32,46]. A summary of the most important study characteristics and outcomes of the 39 included studies stratified by vaccination schedule can be found in Table 1. A more detailed description of the studies is provided in the supplementary data. In 23/39 studies, more than 75% of PLWH were treated with cART [5,^13^,^14^,^24^,^25^,[27], [28], [29], [30],^32^,^33^,^35^,[37], [38], [39], [40], [41],^43^,^44^,[46], [47], [48],[52], [53], [54],56] and in 17/39 studies, the mean/median CD4 count was above 500 cells/mm^3^ [5,^13^,^23^–^25^,^27^,^28^,^35^,^38^,^39^,^43^–^45^,^47^,^48^,^52^,55]. In only 3/39 studies, 100% of patients were treated with cART and had suppressed viral loads, reflecting the current standard of care [58]. Importantly, less than 7 serotypes were analysed in the majority of trials (20/39); and none of the studies assessed all 23 serotypes. The most widely studied serotypes were serotype 6B and serotype 14, both in 25 studies (Table 1).

**Table 1:** Characteristics and outcomes of included studies.

**A) Studies investigating one dose of PPSV23**†								
Study (year)*	**Definition of seroconversion**	**Vaccination schedule**	**Subgroup**	**CD4 count (mean/median) cells/ mm^3^**	**cART**‡**%**	**Seroconversion rate n/N(%)**		
						6B	14	Overall
**Almeida (2009)****[23]**	≥ 2-fold IgG increase; Overall: to ≥ 4 serotypes (70%)	PPSV23		503	45%	24/44(55)	25/44 (57)	11/44 (25)
**Amendola (2002)****[22]**	≥ 2-fold IgG increase	PPSV23		433	40%	4/10 (40)	NR|	NR
			CD4 <200					
			CD4 200–500		52%	9/23 (39)	NR	NR
			CD4 >500		33%	5/24 (21)	NR	NR
			HIV-§	NR	0%	7/20 (35)	NR	NR
Chang (2000) [26]	NR	PPSV23	HIV+	149	50%	NR	NR	NR
			HIV-	NR	NA	NR	NR	NR
**Falco (2006)****[27]**	≥ 2-fold IgG increase Overall: to ≥ 3 serotypes (60%)	PPSV23	HIV+	504	79%	15/112 (13.4)	71/111 (64)	28/112 (25)
			HIV-	NR		11/30 (37)	19/30 (63)	14/30 (47)
Hart (2007) [29]	≥ 4-fold IgG increase	PPSV23	HIV+	409	100%	NR	NR	NR
			HIV-	788	NR	NR		NR
Horster (2010) [30]	≥ 2-fold IgG increase	PPSV23		446	85%	NR	NR	67/98 (68)
Huang (2018) [31]	Opsonic titer ≥ 8	PPSV23	HIV+	350–970 (range)	> 50% (NR)	60/63 (95)	NR	NR
			HIV-	NR	NA	56/56 (100)	NR	NR
Hung (2010) [32]**5-year follow-up data**	≥ 2-fold IgG increase (to any serotype, 25%) **5y**	PPSV23	CD4 <100	45	97%	NR	2/22 (9.1)	**5y** 47/141 (33)
			CD4 100–199	146	100%	NR	12/34 (35)	
			CD4 200–349	263	100%	NR	9/30 (30)	
			CD4 ≥350	457	98%	NR	17/56 (30)	
Kang (2016) [33]	NR	PPSV23		NR	95%	NR	NR	NR
Leggat (2015) [34]	NR	PPSV23	CD4>200 cART-	553	0%	NR	NR	NR
			CD4<200 cART-	126	0%	NR	NR	NR
			CD4<200 cART+	206	100%	NR	NR	NR
			HIV-	NR	0%	NR	NR	NR
MacLennan (2016) [38]	NR	PPSV23	HIV+	500	83%	NR	NR	NR
			HIV-			NR	NR	NR
Payeras (2002) [40]	NR	PPSV23	HIV+ recurrent bacterial infection	242	76%	NR	NR	NR
			HIV+ controls	247	97%	NR	NR	NR
			HIV-	NR	NA	NR	NR	NR
**Rash (2015)****[41]**	≥ 2-fold IgG increase AND ≥1.30 mcg/ml to 70% of serotypes	PPSV23		NR	100%	NR	NR	16/23 (70)
Rodriguez-Barradas (2003) [42]	≥ 2-fold IgG increase OR ≥1.0 mcg/ml for ≥ 2 serotypes (33%)	PPSV23	(cART+) first time	352	100%	NR	NR	25/46 (54)
			(cART+) second time	366	100%	NR	NR	22/41(54)
			(cART-)	274	0%	NR	NR	23/39 (61)
**Rodriguez-Barradas (2015)****[49]**	≥ 2-fold IgG increase AND ≥1.0 mcg/ml; Overall: for 3 serotypes (60%)	PPSV23/Placebo (mo 0, mo 9–12)		303	0%	NR	NR	5/36 (14)
		Placebo/PPSV23 (mo 0, mo 9–12)		470	100%	NR	NR	3/36 (8.3)
**Tasker (2002)****[57]**	≥ 2-fold IgG increase; Overall: for ≥ 2 serotypes (50%)	PPSV23	first time (cART-)	579	0%	9/14 (64)	11/14 (79)	13/14 (93)
			second time (cART+)	274	52%	9/56 (16)	25/56 (45)	25/56 (45)
		Placebo	second time (cART+)	269	45%	0/29 (0)	1/29 (3)	0/29 (0)
**Tsachouridou** **(2015)****[45]**	≥ 2-fold IgG increase	PPSV23	cART+	709	100%	NR	NR	24/35 (69)
			cART-	638	0%			26/31 (84)


**B) Studies investigating PCV**¶**/Multiple PCV doses**								
Study (year)*	**Definition of seroconversion**	**Vaccination schedule**	**Subgroup**	**CD4 count (mean/median) cells/ mm^3^**	**cART**‡**%**	**Seroconversion rate n/N(%)**		
						6B	14	Overall
Bhorat (2015) [25]	NR	3x PCV13 (mo 0,1,2) + PPSV23 (mo 3)		537	96.7%	NR	NR	NR
Glesby (2015) [28]	NR	PCV13+PCV13+PCV13 (0,6,12 mo)		605	95%	NR	NR	NR
**Lu (2012)****[36]**	≥ 2-fold IgG increase AND ≥1.0 mcg/ml Overall: for ≥ **2** (50%)	PCV7 (wk 0)		439	72%	NR	NR	63/107 (59)
		PCV7+PCV7 (wk 0,wk 4)		450	71%	NR	NR	97/113 (86)
Cheng (2016) [46] 5**-years follow-up Lu (2012)**	≥ 2-fold IgG increase Overall: to ≥ 2 serotypes (50%)	PCV7 (wk0)		407	77%	57/102 (56)	73/102 (72)	63/102 (62)
		PCV7+PCV7 (wk0,4)		446	81%	56/103(54)	79/103 (77)	78/103 (76)
Rossheim (2016) [43]	NR	PCV13	(PPSV23 1–3 years earlier)	657	100%	NR	NR	NR
			(PPSV23 >3 years earlier)	602	100%	NR	NR	NR
Song (2019) [44]	≥4-fold increase of OPA titer	PCV13	CD4 >349	579	100%	25/34 (74)		NR
		PCV13	CD4 <350	200	100%	20/33 (61)		NR


**C) Studies investigating PCV + PPSV23 combined**								

Study (year)*	**Definition of seroconversion**	**Vaccination schedule**	**Subgroup**	**CD4 count (mean/median) cells/ mm^3^**	**cART**‡**%**	**Seroconversion rate n/N(%)**		
						6B	14	Overall
**Deloria-Knoll (2006)****[50]**	NR	PCV 7 + PPSV23 (mo 0, mo 6)		402	11%	23/63 (37)	40/63 (64)	29/62 (47)
**Farmaki (2018)****[5]**	≥ 2-fold IgG increase	PCV13+PPSV23 (0,12 mo)		530	100%	NR	40/40 (100)	NR
**Sögaard (2010)****[47]**	≥ 2-fold IgG increase AND ≥1.0 mcg/ml; Overall: for ≥ 5 PCV7 serotypes (70%)	PCV7/PCV7/PPSV23 (mo 0, 3,9)	No adjuvans	605	79%	32/47 (68)	34/47 (72)	24/47 (51)
			CPG7909 adjuvans	673	79%	35/42 (83)	36/42 (85)	36/41 (88)


**D) Studies comparing PCV (or multiple PCV doses) to PPSV23**								

Study (year)*	**Definition of seroconversion**	**Vaccination schedule**	**Subgroup**	**CD4 count (mean/median) cells/ mm^3^**	**cART**‡**%**	**Seroconversion rate n/N(%)**		
						6B	14	Overall
**Crum-Cianflone (2010)****[48]**	≥ 2-fold IgG increase AND ≥1.0 mcg/ml; Overall: to ≥ **2** serotypes (50%)	PPSV23	HIV+	513	77%	NR	28/ 64 (44)	23/63 (36)
		PCV7	HIV+	533	85%	NR	61/120 (51)	68 /120 (57)
			HIV-	NR	0%	NR	18/25 (72)	22/25 (88)
**Feikin (2001)****[51]**	≥ 2-fold IgG increase	PCV7+PCV7 (0,8wk)		378	67%	*6/15 (40) from graph*	*4/15 (27) from graph*	NR
		PCV7+PPSV23 (0,8wk)		434	28%	*8/18 (44) from graph*	*7/18 (39) from graph*	NR
		Placebo+PPSV23 (0,8wk)		508	31%	*3/16 (19) from graph*	*7/16 (44) from graph*	NR
		2 doses of placebo (0,8wk)		403	44%	NR	NR	NR
Ho (2013) [52]	≥ 4-fold IgG increase	PPSV23+Placebo (mo 0, mo 2)		548	73%	7/89 (7.9)	35/89 (39)	NR
		PCV7/Placebo (mo 0, mo 2)		545	76%	27/91 (30)	50/91 (56)	NR
		PCV7/PPSV23 (mo 0, mo 2)		492	81%	27/91 (30)	42/91 (46)	NR
**Lombardi (2016)****[13]**	≥ 2-fold IgG increase; Overall for ≥ 9 PCV serotypes (70%)	PCV13+PCV13 (wk 0, 8)		591	98%	27/46 (59)	28/46 (61)	10/46 (22)
		PPSV23 (wk0)		639	100%	20/49 (41)	33/49 (67)	14/49 (29)
Belmonti (2019) [24] 5**-years follow-up Lombardi (2016)**	≥ 2-fold IgG increase; Overall: to ≥ ≥9 serotypes (70%)	PCV13+PCV13 (wk 0, 8)		591	98%	17/42(40)	13/42 (31)	2/42 (4.8)
		PPSV23 (wk0)		639	100%	9/49(18)	14/49 (29)	3/49 (6.1)
Lu (2014) [35]	≥ 2-fold IgG increase AND ≥1.0 mcg/ml; Overall: for ≥ 2 serotypes (50%)	PPSV23 (wk 0)		519	100%	NR	NR	11/97 (11)
		PCV7 (wk 0)		565	90%	NR	NR	16/36 (44)
		PCV7+PCV7 (wk 0,wk 4)		479	91%	NR	NR	28/39 (72)
Lu (2013) [37]	≥ 2-fold IgG increase AND ≥1.0 mcg/ml; Overall: for ≥ 2 serotypes (50%)	PPSV23		408	100%	NR	NR	21/104 (20)
		PCV7		403	72%	NR	NR	39/104 (38)
**Slayter (2013)****[14]**	≥ 2-fold IgG increase; Overall: for **4** serotypes (57%)	PCV7	(immediate | delayed)	(82|77)	97%	7/19(37) 7/10 (70)	10/19 (53) 7/10 (70)	7/19 (37) | 7/10 (70)
		PPSV23	(immediate | delayed)	(64|90)	100%	10/18 (56) 6/17 (35)	8/18 (44) 11/17 (65)	11/18 (61) | 11/17 (65)
NCT02717494 (2020) [56]	≥ 2-fold IgG increase for at least 1 serotype	PCV10		596	100%	NR	NR	112/114 (98)
		PPSV23		585	100%	NR	NR	106/110 (96)
		Placebo		564	100%	NR	NR	7/113 (6.2)


**E) PPSV23 versus PCV+PPSV23 combined**								

Study (year)*	**Definition of seroconversion**	**Vaccination schedule**	**Subgroup**	**CD4 count (mean/median) cells/ mm^3^**	**cART**‡**%**	**Seroconversion rate n/N(%)**		
						6B	14	Overall
**Feikin (2001)****[51]**	≥ 2-fold IgG increase	PCV7+PCV7 (0,8wk)		378	67%	*6/15 (40) from graph*	*4/15 (27) from graph*	NR
		PCV7+PPSV23 (0,8wk)		434	28%	*8/18 (44) from graph*	*7/18 (39) from graph*	NR
		Placebo+PPSV23 (0,8wk)		508	31%	*3/16 (19) from graph*	*7/16 (44) from graph*	NR
		2 doses of placebo (0,8wk)		403	44%	NR	NR	NR
Ho (2013) [52]	≥ 4-fold IgG increase	PPSV23+Placebo (mo 0, mo 2)		548	73%	7/89 (7.9)	35/89 (39)	NR
		PCV7/Placebo (mo 0, mo 2)		545	76%	27/91 (30)	50/91 (56)	NR
		PCV7/PPSV23 (mo 0, mo 2)		492	81%	27/91 (30)	42/91 (46)	NR
**Lesprit (2007)****[53]**	≥ 2-fold IgG increase AND ≥1.00 mcg/ml; Overall: for ≥ 5 PCV7 serotypes (70%)	PCV7+PPSV23 (wk 0, wk 4)		351	88%	57/105 (54)	74/105 (70)	62/105 (59)
		PPSV23 (wk4)		350	86%	53/103 (51)	70/103 (68)	41/103 (40)
**Ohtola (2016)****[39]**	≥ 2-fold increase AND ≥1.00 mcg/ml for 0, **1**, 2 serotypes	PPSV23	HIV+	652	100%	NR	NR	12/22 (55)
		PCV13+PPSV23 (wk 0, 8)	HIV+	717	100%	NR	NR	11/15 (73)
			HIV-	NR	NA	NR	NR	12/14 (86)
**Peñaranda (2010)****[54]**	≥ 2-fold increase AND ≥1.00 mcg/ml to ≥ 4 serotypes (57%)	PCV7/PPSV23 (wk0, wk4)		368	98%	31/98 (32)	49/98 (50)	NR
		PPSV23 (wk 0)		351	91%	30/100 (30)	49/100(49)	NR
**Sadlier (2016)****[55]**	≥ 2-fold increase AND ≥1.0 mcg/ml; Overall: for ≥ 7 serotypes (58%)	PCV13+PPSV23 (wk 0, 4)		447	52%	NR	NR	16/26 (62)
		PPSV23 (wk 4)		572	40%	NR	NR	11/28 (39)

⁎ Studies printed in bold were included in the meta-analysis. For additional information on study design, methodology, patient characteristics and outcomes of individual studies we refer to the supplementary material.

† PPSV23 = 23-valent pneumococcal polysaccharide vaccine.

‡ cART= combination antiretroviral treatment, defined as a combination of at least antiretroviral drugs.

§ HIV= human Immunodeficiency virus.

| NR = not reported.

¶ PCV= pneumococcal conjugate vaccine.

### 3.2 Risk of bias assessment

Results of the risk of bias assessment can be found in Table 2 (cohort studies) and Table 3 (RCTs). Of the cohort studies, only five were of good quality, ten were of fair quality, and 13 were of poor quality. Important flaws in cohort studies were questionable representativeness of the cohort (*n* = 16); lack of a representative HIV-negative control cohort (*n* = 22); or failure to adjust for important confounding factors such as age, comorbidities and CD4-cell count in comparative analyses (*n* = 16).

**Table 2:** Risk of bias assessment cohort studies*.

Study	**Selection**				**Comparability**	**Outcome**			
	Representativeness of exposed cohort (max: ✪) a	Selection of non-exposed cohort (max: ✪)b	Ascertainment of exposure (max: ✪) c	Outcome was not present at start of study (max: ✪) d	Comparability of cohorts on the basis of the design or analysis (max ✪✪) e	Assessment of outcome (max: ✪) f	Follow-up long enough(max: ✪) g	Adequacy of follow-up of cohorts (max: ✪) h	**Overall**
Almeida (2009) [23]	▪	▪	✪	✪	▪	▪	▪	✪	**Poor**
Amendola (2002) [22]	▪	✪	✪	✪	✪	✪	✪	▪	**Good**
Belmonti (2019) [24]	✪	▪	✪	✪	✪✪	✪	✪	✪	**Good**
Bhorat (2015) [25]	✪	▪	✪	✪	▪	✪	✪	▪	**Fair**
Chang (2000) [26]	▪	▪	✪	✪	✪	▪	✪	▪	**Poor**
Cheng (2016) [46]	▪	▪	✪	✪	✪✪	✪	✪	▪	**Fair**
Falco (2006) [27]	▪	✪	✪	✪	✪	✪	✪	✪	**Good**
Farmaki (2018) [5]	✪	▪	✪	✪	✪	▪	✪	✪	**Fair**
Glesby (2015) [28]	✪	▪	✪	✪	▪	✪	✪	▪	**Poor**
Hart (2007) [29]	▪	✪	✪	✪	✪	▪	✪	▪	**Poor**
Horster (2010) [30]	✪	▪	✪	✪	✪✪	✪	✪	✪	**Fair**
Huang (2018) [31]	✪	✪	✪	✪	▪	▪	✪	✪	**Poor**
Hung (2010) [32]	▪	▪	✪	✪	✪	✪	✪	▪	**Fair**
Kang (2016) [33]	✪	▪	✪	▪	▪	✪	▪	✪	**Poor**
Leggat (2015) [34]	▪	▪	✪	▪	▪	▪	✪	✪	**Poor**
Lombardi (2016) [13]	✪	▪	✪	✪	✪	✪	✪	✪	**Good**
Lu (2012) [36]	▪	▪	✪	✪	✪	✪	✪	✪	**Fair**
Lu (2014) [35]	✪	▪	✪	✪	✪		✪	▪	**Poor**
Lu (2013) [37]	▪	▪	✪	✪	✪	✪	✪	▪	**Fair**
MacLennan (2016) [38]	✪	✪	✪	✪	✪	✪	✪	▪	**Good**
Ohtola (2016) [39]	▪	✪	✪	✪	▪	✪	✪	✪	**Poor**
Payeras (2002) [40]	▪	▪	✪	✪	▪	▪	✪	✪	**Poor**
Rash (2015) [41]	▪	▪	✪	✪	▪	✪	✪	✪	**Poor**
Rodriguez-Barradas (2003) [42]	▪	▪	✪	✪	▪	✪	✪	✪	**Poor**
Rossheim (2016) [43]	✪	▪	✪	▪	✪	✪	✪	✪	**Fair**
Song (2019) [44]	▪	▪	✪	✪	✪	✪	▪	✪	**Fair**
Tasker (2002) [57]	▪	▪	✪	✪	▪	✪	✪	✪	**Poor**
Tsachouridou (2015) [45]	▪	▪	✪	✪	✪	✪	✪	✪	**Fair**

⁎ An explanation of the scoring system can be found in Supplementary File 2.

**Table 3:** Risk of bias assessment randomised clinical trials.

	Selection bias		Performance bias	Detection bias	Attrition bias	Reporting bias		**Overall assessment**
**Study**	**Random sequence generation**	**Allocation concealment**	**Blinding of participants and researchers**	**Blinding of outcome assessment**	**Incomplete outcome data**	**Selective reporting**	Other bias*	
**Sögaard (2010)****[47]**	**Low risk of bias**	**Low risk of bias**	**Low risk of bias**	**Low risk of bias**	**Low risk of bias**	**Low risk of bias**	**Unclear risk**	Good quality
**Crum-Cianflone (2010)****[48]**	**Unclear risk of bias**	**Unclear risk of bias**	**Low risk of bias**	**Low risk of bias**	**Unclear risk of bias**	**High risk of bias**	**Low risk**	Poor quality
**Rodriguez-Barradas (2015)****[49]**	**Low risk of bias**	**Low risk of bias**	**Low risk of bias**	**Low risk of bias**	**Low risk of bias**	**High risk of bias**	**Low risk**	Fair quality
**Deloria-Knoll (2006)****[50]**	**Low risk of bias**	**Low risk of bias**	**Low risk of bias**	**Low risk of bias**	**Low risk of bias**	**Low risk of bias**	**Unclear risk**	Good quality
**Feikin (2001)****[51]**	**Unclear risk of bias**	**Unclear risk of bias**	**Low risk of bias**	**Low risk of bias**	**High risk of bias**	**Unlcear risk of bias**	**Unclear risk**	Poor quality
**Ho (2013) [52]**	**Low risk of bias**	**Unclear risk of bias**	**Low risk of bias**	**Low risk of bias**	**Unclear risk of bias**	**Low risk of bias**	**Low risk**	Fair quality
**Lesprit (2007)****[53]**	**Low risk of bias**	**Low risk of bias**	**Low risk of bias**	**Low risk of bias**	**Low risk of bias**	**High risk of bias**	**Unclear risk**	Poor quality
**Peñaranda (2010)****[54]**	**Unclear risk of bias**	**Unclear risk of bias**	**Low risk of bias**	**Low risk of bias**	**Low risk of bias**	**High risk of bias**	**Low risk**	Poor quality
**Sadlier (2016)****[55]**	**Low risk of bias**	**Unclear risk of bias**	**Low risk of bias**	**Low risk of bias**	**Unclear risk of bias**	**Unlcear risk of bias**	**Unclear risk**	Poor quality
**Slayter (2013)****[14]**	**Unclear risk of bias**	**Unclear risk of bias**	**Low risk of bias**	**Low risk of bias**	Low risk of bias	Low risk of bias	**Unclear risk**	Fair quality
**Tasker (2002)****[57]**	**Low risk of bias**	**Low risk of bias**	**Low risk of bias**	**Low risk of bias**	**Low risk of bias**	**Unlcear risk of bias**	**High risk**	Poor quality
**NCT02717494 (2020)****[56]**	**Unclear risk of bias**	**Unclear risk of bias**	**Low risk of bias**	**Low risk of bias**	**Low risk of bias**	**Low risk of bias**	**Unclear risk**	Fair quality

⁎ Unclear if important information on analysis, patient population or results was not available in manuscript; high risk if the statistical analysis may have introduced bias.

Of the RCTs, 2 were of good quality, 4 were of fair quality and 6 were of poor quality. High risk of reporting bias was the most important issue, followed by attrition bias and insufficient explanation of the random sequence generation and allocation concealment (selection bias).

### 3.3 Seroconversion rates after pneumococcal vaccination

Overall SCRs, defined as a response to >50% of serotypes, varied widely between studies. For PPSV23 alone, overall SCRs ranged from 8.3%−93% when all studies were considered, from 26 to 70% in studies with >75% cART coverage, and from 25 to 55% in studies with median CD4-cell counts >500 cells/mm^3^. For PCV, overall SCRs ranged from 22 to 86% when all studies were considered, from 22 to 70% in studies with >75% cART coverage, and from 25 to 55% in studies with median CD4 counts >500 cells/mm^3^. For PCV/PPSV23 combinations, SCRs ranged from 51 to 100%. This range was the same if only studies with >75% cART coverage and with CD4-cell counts >500 cells/mm^3^ were considered. Overall SCRs in HIV-negative individuals (to PPSV, PCV or a combination) ranged between 86 and 100%.

**Fig. 2:**
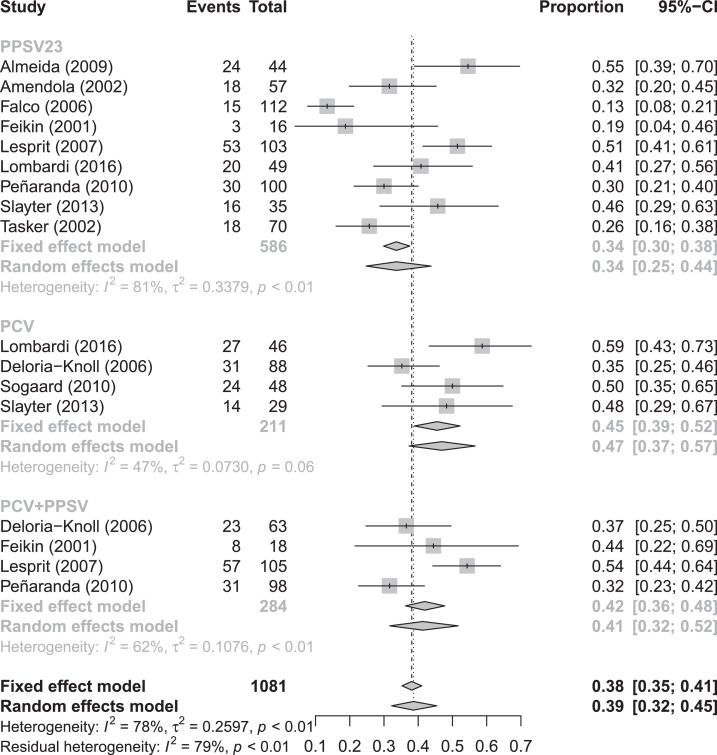
Forest plot of seroconversion rates to serotype 6B

**Fig. 3:**
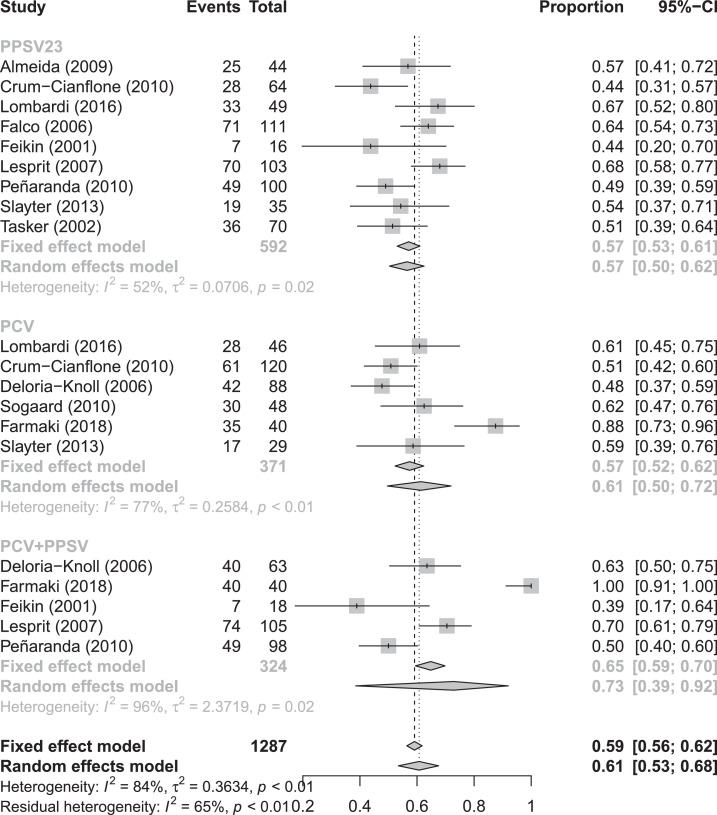
Forest plot of seroconversion rates to serotype 14.

**Fig. 4:**
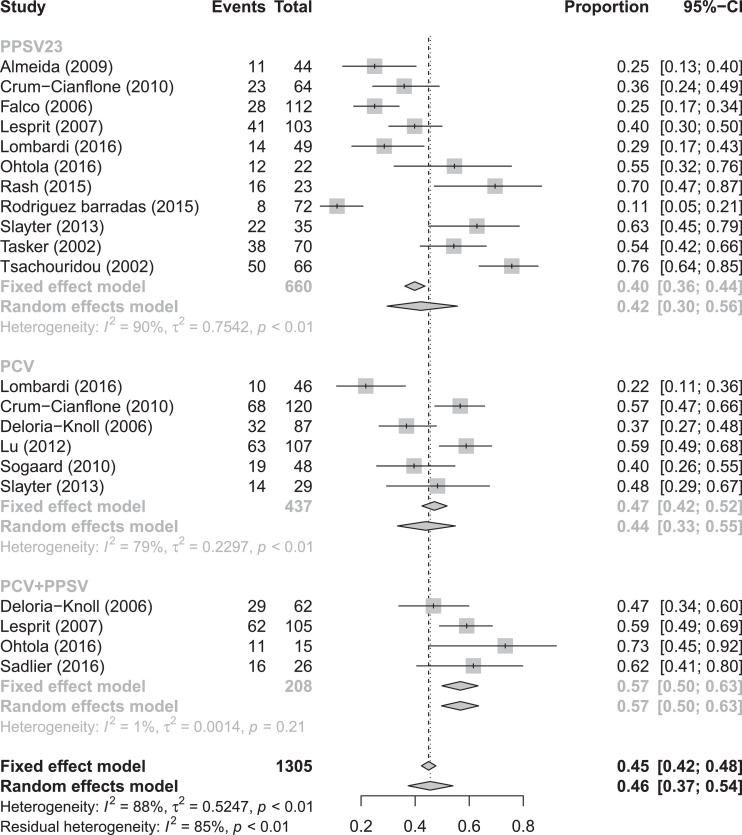
Forest plot of overall seroconversion rates.

In random effects meta-analysis, pooled SCRs to serotype 6B were 34% (95% CI 25–44%), 47% (95% CI 37–57%), and 41% (95% CI 32–52%) for PLWH who received PPSV, PCV or a combination of PCV/PPSV, respectively (Fig. 2). Pooled SCRs rates to serotype 14 were 57% (95% CI 50–62%), 61% (95% CI 50–72%), and 73% (95% CI 39–92%) for PLWH that received PPSV, PCV and a combination of PCV/PPSV, respectively (Fig. 3). Pooled overall SCRs were 42% (95% CI 30–56%), 44% (95% CI 33–55%) and 57% (95%CI 50–63%) for PLWH who received PPSV, PCV or a combination of PCV/PPSV, respectively (Fig. 4). A high degree of heterogeneity was observed when SCRs were pooled, with I^2^ ranging up to 96%. Neither different results nor a lower degree of heterogeneity were observed when we performed subgroup analyses based on CD4 count, study quality and cART use (data not shown).

### 3.4 Comparisons of different vaccination strategies

Seven of the nine individual studies that compared PCV to PPSV23 reported a higher GMC or SCR to at least 1 serotype after PCV; while two other studies found an equal response**.** Only one study found an additional advantage of multiple PCVs (Table 4). Of note, these studies only investigated PCV serotypes and did not take the additional 10 serotypes unique to PPSV23 into consideration. Among six studies that compared PPSV23 to the combined schedule of PCV/PPSV23, four found a better response when both vaccinations had been administered, while two found comparable immunogenicity (Table 5). Regarding multiple doses of PCV, one study reported a better SCR when two doses of PCV 4 weeks apart were administered compared to a single PCV [36]. and this difference in SCR persisted 5 years after vaccination [46]. In contrast, four trials showed only marginal or absent IgG increases or improved SCR when a second or third dose of PCV was administered [13,^25^,^28^,^51^. When responses to pneumococcal vaccination in PLWH were compared to HIV-negative controls (*n* = 8 studies), all studies found better responses in HIV-negative controls (Table 6).

**Table 4:** Studies comparing PPSV* to one or multiple PCV† doses.

Study	Higher response PCV versus PPSV	Additional response multiple PCVs
Belmonti (2019) [24]	Yes	NA‡
Crum-Cianflone (2010) [48]	Yes	NA
Ho (2013) [52]	Yes	NA
Feikin (2001) [51]	Yes	No
Lombardi (2016) [13]	Yes	No
Lu (2014) [35]	Yes	NA
Lu (2013) [37]	Yes	Yes
Slayter (2013) [14]	No	NA
NCT02717494 (2020) [56]	No	NA

⁎ PPSV= pneumococcal polysaccharide vaccine.

† PCV= pneumococcal conjugate vaccine.

‡ NA= not applicable, this was not investigated.

**Table 5:** Studies comparing PPSV* to PPSV/ PCV† combined.

Study	Higher response PCV/PPSV
Feikin (2001) [51]	Yes
Ho (2013) [52]	Yes
Sadlier (2016) [55]	Yes
Lesprit (2007) [53]	Yes
Ohtola (2016) [39]	No
Peñaranda (2010) [54]	No

⁎ PPSV= pneumococcal polysaccharide vaccine.

† PCV= pneumococcal conjugate vaccine.

**Table 6:** Studies comparing pneumococcal vaccines in PLWH* versus HIV-negative individuals.

Study	Higher response in HIV-negative individuals
PPSV†	
Falco (2006) [27]	Yes
Hart (2007) [29]	Yes
Huang (2018)	Yes
Leggat (2015) [34]	Yes
Maclennan (2016) [38]	Yes
Payeras (2002) [40]	Yes
PCV‡	
Crum-Cianflone (2010) [48]	Yes
PCV/PPSV combined	
Ohtola (2016) [39]	Yes

⁎ PLWH=People living with human immunodeficiency virus.

† PPSV= pneumococcal polysaccharide vaccine.

‡ PCV= pneumococcal conjugate vaccine.

**Fig. 5:**
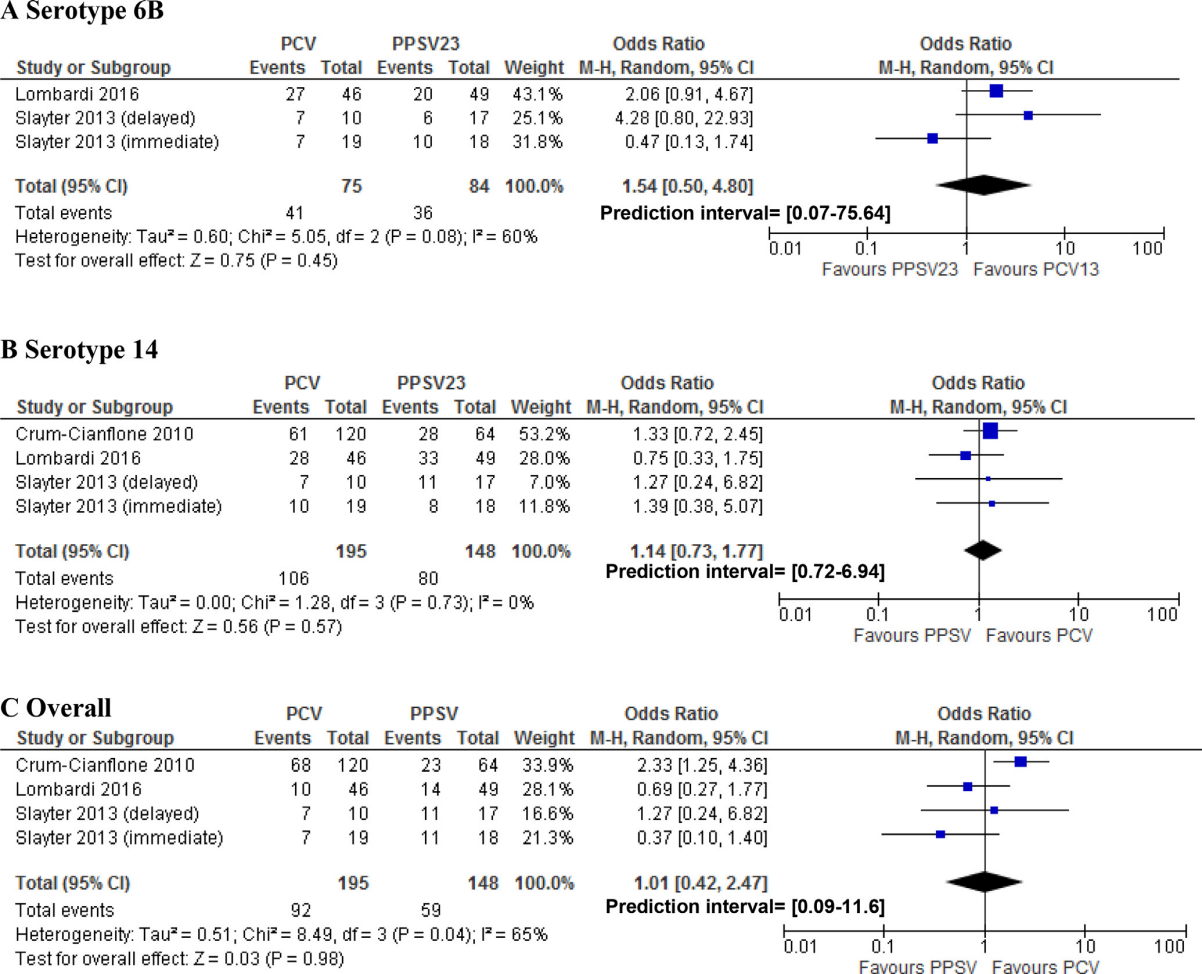
Forest plot of studies comparing seroconversion rates for the pneumococcal conjugated vaccine (PCV) versus the pneumococcal polysaccharide vaccine (PPSV).

**Fig. 6:**
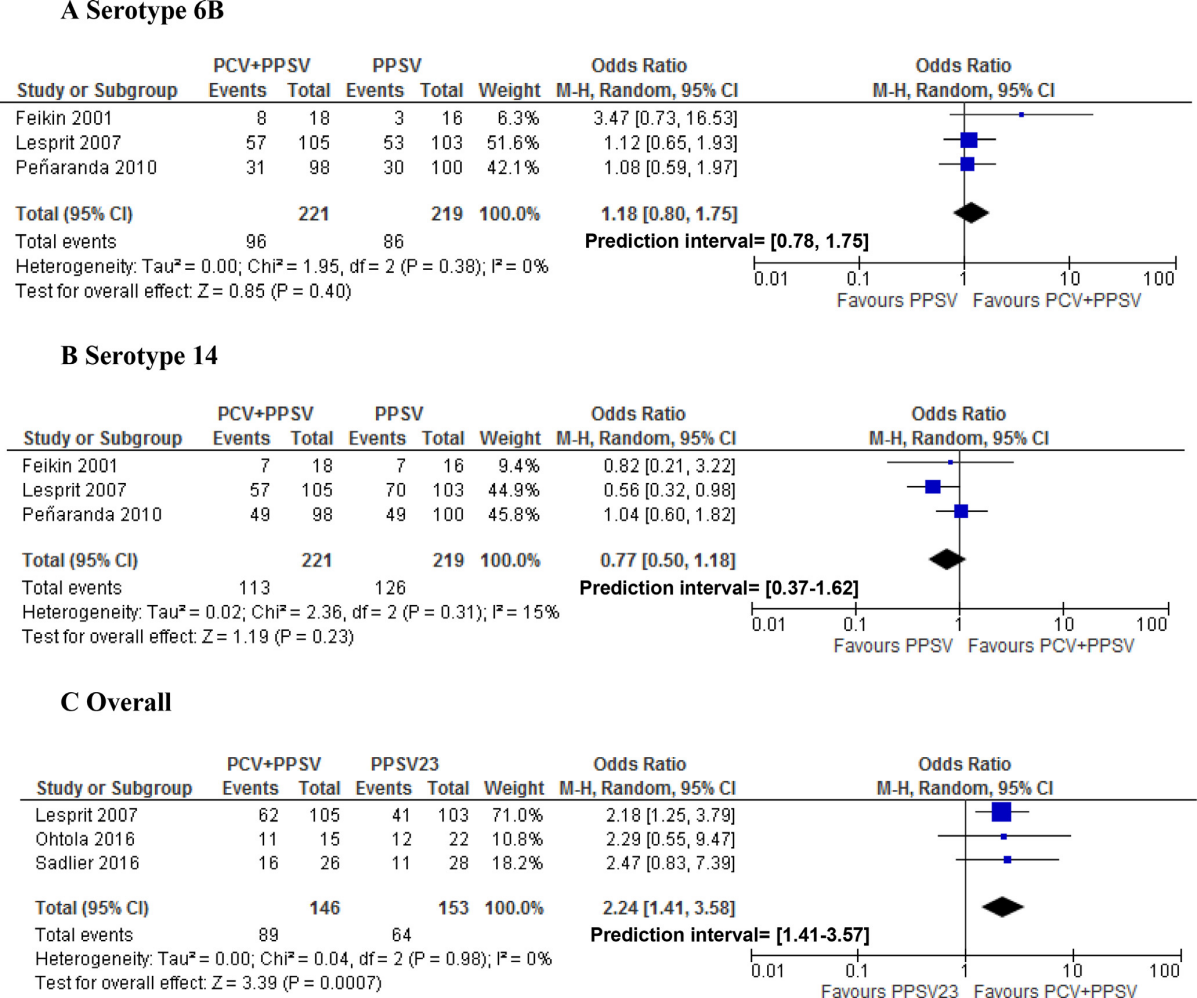
Forest plot of studies comparing seroconversion rates for the combined pneumococcal vaccination schedule (PCV+PPSV)  versus the pneumococcal polysaccharide vaccine (PPSV) alone.

In a random-effects meta-analysis, there were no statistically significant differences in SCRs for serotypes 6B and 14 when comparing PPSV23 to PCV (Fig. 5), or to a combination of PCV/PPSV23 (Fig. 6). The overall SCR was the same for PPSV23 compared to PCV (OR 1.01 95% CI 0.42–2.47; Fig. 5). However, when comparing overall SCRs, PLWH who received a combination of PCV/PPSV23 were more likely to seroconvert than PLWH who only received PPSV23 (OR 2.24 95% CI 1.41- 3.58; Fig. 6).

### 3.5 Geometric mean antibody concentrations

GMCs of pre-and post-vaccination IgG were reported in (25/39) studies, and varied widely. Pre-vaccination GMCs for serotype 6B ranged between 0.14 to 5.99 µg/ml, and post vaccination GMCs ranged between 0.43 and 8.5 µg/ml. For serotype 14, pre-vaccination GMCs ranged between 0.25 and 7.3 µg/ml, and post-vaccination GMCs ranged between 0.80 and 27.8 µg/ml.

In a random-effects meta-analysis comparing log-transformed GMCs of serotype 6B and 14, we found no significant differences between PLWH who received PCV compared to PPSV23, except a higher baseline (pre-vaccination) IgG level for serotype 14 among those who received PPSV23 (Supplementary material fig. 3). When comparing PPSV23 alone versus a combined schedule of PCV/PPSV23, we found no statistically significant differences in log-transformed GMCs, but a trend towards a higher post-vaccination level for serotype 14 in PLWH who received the combined schedule of PCV/PPSV23 (Supplementary material fig. 4).

### 3.6 Influence of CD4-count and cART status or viral load on vaccination response

Vaccination at higher CD4 cell counts improved immunogenicity in 8/21 studies (Table 7), while suppressed viral load/cART use improved immunogenicity in 7/18 studies (Table 8). Of note, most studies that reported a positive effect of higher CD4-cell count on vaccination response investigated schedules with at least one dose PCV (5/8).

**Table 7:** Studies reporting the effect of CD4-cell count on immune response.

Study	Vaccination schedule	Better response at higher CD4 count	Cut-off CD4-cell count associated with higher response in cells/mm^3^
Amendola (2002) [22]	PPSV*	No	NA‡
Cheng (2016) [46]	PCV[†] / 2x PCV	Yes	NA
Crum-Cianflone (2010) [48]	PCV/PPSV	No	NA
Falco (2006) [27]	PPSV	No	NA
Feikin (2001) [51]	PCV+PPSV/ 2xPCV/ PPSV	No	NA
Hart (2007) [29]	PPSV	No	NA
Horster (2010) [30]	PPSV	No	NA
Hung (2010) [32]	PPSV	Yes	100
Leggat (2015) [34]	PPSV	Yes	200
Lesprit (2007) [53]	PPSV / PCV+ PPSV	No	NA
Lombardi (2016) [13]	PPSV / 2x PCV	No	NA
Lu (2012) [36]	PCV/ 2x PCV	No	NA
Lu (2013) [37]	PCV / PPSV	No	NA
Lu (2014) [35]	PCV/PPSV / 2xPCV	Yes	350
Maclennan (2016) [38]	PPSV	No	NA
Rodriguez-Barradas (2003) [42]	PPSV	Yes	500
Rodriguez-Barradas (2015) [49]	PPSV	No	NA
Peñaranda (2010) [54]	PPSV	No	NA
Rossheim (2016) [43]	PCV	Yes	500
Song (2019) [44]	PCV	Yes	350
Sogaard (2010) [47]	2xPCV+PPSV	Yes	500

⁎ PPSV= pneumococcal polysaccharide vaccine.

† PCV= pneumococcal conjugate vaccine.

‡ NA= not applicable, this was not investigated.

**Table 8:** Studies reporting the effect of suppressed viral and/or combined antiretroviral treatment use load on immune response.

Study	Vaccination schedule	Surpressed VL*/cART† better response
Cheng (2016) [46]	PCV‡ / 2x PCV	Yes
Crum-Cianflone (2010) [48]	PCV/PPSV§	No
Falco (2006) [27]	PPSV	Yes
Feikin (2001) [51]	PCV+PPSV/ 2xPCV/ PPSV	No
Hart (2007) [29]	PPSV	No
Horster (2010) [30]	PPSV	No
Hung (2010) [32]	PPSV	Yes
Leggat (2015) [34]	PPSV	No
Lesprit (2007) [53]	PPSV / PCV+ PPSV	No
Lombardi (2016) [13]	PPSV / 2x PCV	No
Lu (2012) [36]	PCV/2x PCV	No
Lu (2013) [37]	PCV/ PPSV	Yes
Lu (2014) [35]	PCV/PPSV	Yes
Peñaranda (2010) [54]	PPSV	No
Rodriguez-Barradas (2003) [42]	PPSV	No
Rodriguez-Barradas (2015) [49]	PPSV	No
Sogaard (2010) [47]	2xPCV+PPSV	Yes
Tsachouridou (2015) [45]	PPSV	Yes

⁎ cART = combined antiretroviral treatment.

† VL = viral load.

‡ PCV= pneumococcal conjugate vaccine.

§ PPSV= pneumococcal polysaccharide vaccine.

### 3.7 Timing of vaccination

With respect to the timing of vaccination, three studies investigated the difference between immediate pneumococcal vaccination upon HIV diagnosis versus delayed pneumococcal vaccination until after six months after initiation of cART [14,^34^,49]. Two of these studies reported no immunological benefits of delaying PPSV23 [34,49] By contrast Slayter et al. showed that delaying vaccination until immune reconstitution significantly improved vaccination responses, with the strongest positive effect for the immune response to PCV. Importantly, this study had only included individuals with CD4 cell counts below 200 cells/mm [3,14].

### 3.8 Long-term immunogenicity

No studies reported on the long-term immunogenicity of a combination of PCV/PPSV23. Three studies investigated long term immunogenicity of either PPSV23 or one or two doses of PCV, five years after initial vaccination [24,^32^,46]. All were follow-up studies of the initial immunogenicity trials that were also included in this review (Table 1). Two studies reported on PPSV23 and showed a significant decay in antibody levels five years after vaccination, particularly when vaccinated had occurred at lower CD4-counts; one study showed a decrease in overall SCRs from 29% to 6.1%, while the second study showed that only 33% maintained a response to one out of three serotypes [24,32]. For PCV, results varied in two available studies. In the first study, SCRs dropped from to 22% to 4.8% [24] while in the second study high SCRs and SPRs persisted five years after initial vaccination; with a slight advantage for the group that received two PCV vaccinations instead of one (SCR 76% versus 62%) [46]. Of note, this study reported much higher initial SCRs compared to the first study.

## 4 Discussion

Although immunogenicity varied per vaccine type, overall we found that pneumococcal vaccines are immunogenic in PLWH. Serotype 6B was less immunogenic than serotype 14, which was previously reported in healthy individuals [59]. Comparison of our results with those of recent studies in other immunocompromised individuals yielded similar responses to pneumococcal vaccination in PLWH (overall SCR 57%) and patients using immunosuppressive drugs for auto-immune diseases (overall SCR 59%) [60,61]. That notwithstanding, pneumococcal vaccination responses in PLWH are lower than in healthy individuals, also after exclusion of studies from pre-cART eras. This was previously reported for other vaccines, including hepatitis A, B, and influenza [62,63], and may be the consequence of persistent chronic humoral and cellular immunodeficiency, despite early cART initiation and recovery of T-cell numbers [45,64]. It is important to note that even though studies before 2000 were excluded, only three studies included in this review described cohorts of HIV patients, with 100% cART coverage and suppressed viral loads, reflecting the current standard of care [5,^43^,^56^,58]. In HIV patients who initiate cART immediately after HIV diagnosis, following the current recommendations, better vaccination responses are expected, since this recommended approach reduces immunodeficiency and averts immunological dysfunction caused by chronic inflammation [56].

With respect to the different vaccination schedules, we did not find a statistically significant difference in immunogenicity between PCV and PPSV23. However, caution must be applied here, because the statistical power of some of the comparisons made was low and a clinically relevant difference cannot be ruled out. We did find a statistically significant advantage in overall immunogenicity for the combined vaccination schedule of PCV followed by PPSV23, compared to PPSV23 alone (SCR 57% versus 42%). These findings support the current guidelines, recommending the combined vaccination schedule in PLWH [7]. In HIV-negative individuals, PCV is often more immunogenic than PPSV, due to the advantage of T-cell activation by PCV [65]. We found no statistically significant advantage for PCV in PWLH, which may be caused by HIV mediated T-cell dysfunction. The same was observed in a meta-analysis of individuals using predominantly T-cell affecting immunosuppressive drugs; this analysis even showed higher SCRs after PPSV23 than after PCV [61]. With other vaccines, such as hepatitis A vaccine, additional doses have shown to result in better vaccination responses in PLWH [62]. However, in most studies among PLWH, multiple doses of PCV only resulted in marginal increases in IgG levels or even comparable IgG levels compared to the first PCV dose, and therefore multiple PCV doses are not likely to be cost-effective [25,28]. In addition, PPSV23 has the important advantage that it includes ten serotypes additional to PCV13, which is often overlooked in studies comparing the immunogenicity of PPSV23 versus PCV. Multiple doses of PPSV23 are not recommended due to hyporesponsiveness associated with PPSV23 revaccination at time intervals < 1year [67].

One aim of this review was to determine if the response to vaccination differs between subgroups of PLHW based on CD4-cell count, viral load and/or cART use. Here, the data were inconsistent. Most studies that reported a detrimental effect of lower CD4-cell counts on the response to pneumococcal vaccination investigated schedules with at least one dose of PCV. This corresponds with the immunology of T-cell dependent versus T-cell independent vaccines, highlighting that the advantage of a conjugated vaccine may be irrelevant during T-cell dysfunction [65]. Elaborating on that, delaying PCV vaccination until after immunological recovery of the CD4 T-cell count above 200 cells/mm^3^ in recently diagnosed PLWH is beneficial for the vaccination response and should therefore be recommended [14]. This however does not apply for PPSV23, as was reflected by Leggat et al. [34] and Rodriguez-Barradas et al. [34,49]

The durability of the immune response following pneumococcal vaccination is an important issue, even more so as life expectancy of PLWH under cART has nowadays approximated that of HIV-negative counterparts. In addition, the risk of pneumococcal infections increases at more advanced age [1]. With respect to the durability of the immune response, PCV is often thought to be superior, because PPSV23 does not induce immunologic memory [5,65]. Unfortunately, no studies evaluated the long-term immunogenicity of the combination of PCV/PPSV23 among PLWH. A decay in antibody levels was reported for both PCV and PPSV23, but to a lesser extent when two doses instead of one dose of PCV had been administered [24,^32^,46]. A previous systematic review on long-term immunogenicity of vaccines in PLWH reported that for PPSV23, the decrease in antibody concentrations were either similar (four studies) or more rapid (two studies) compared to HIV-negative individuals. Beyond five years, antibody concentrations had dropped below the cut-off values for most serotypes [66]. Based on these findings, guidelines currently advise a PPSV23 booster dose five years after the initial vaccination schedule [6,7]. It is not recommend to routinely check pneumococcal antibody levels, although this may be local practice in some clinics. We did not find data on the effect of revaccination of PLWH who had not responded to initial vaccination. We emphasize that PPSV23 should not be repeated before one year after the last dose, because of the mentioned risk of hyporesponsiveness, due to memory B-cell depletion. This does not apply to conjugated vaccines [67,68].

In this review, we aimed to provide a comprehensive analysis of the immunogenicity of pneumococcal vaccines in PLWH the era of advanced cART. An important discussion is whether immunogenicity correlates with protection against disease. In adults, no studies of good quality have been conducted on clinical efficacy of pneumococcal vaccines in PLWH on cART [69]. The only RCTs available were performed in sub-Sahara Africa in predominantly untreated HIV patients and with poor immunological status [70,71]. To establish scientific proof of clinical vaccine efficacy against pneumococcal disease, large sample sizes are required, which, for PLWH, do not seem realistic to achieve [10]. Therefore, immunogenicity data, as next-best proxy for vaccine efficacy, remain the cornerstone for vaccination recommendations in immunocompromised individuals.^6^ The WHO cut-off for protection against pneumococcal disease in children was established at 0.35 µg/ml [12]. However, a recent study showed that serotype-specific correlates of protection vary widely, and – in children - ranged between 0.16 and 2.83 µg/ml. [11] When considering fold-change from baseline in the definition for response, the American Academy of Allergy, Asthma and Immunology (AAAAI), defines seroconversion as *a* ≥ 4-fold increase in IgG from baseline. However, in HIV infected adults with higher baseline IgG levels, *a* ≥ 2-fold increase may be regarded as appropriate [21,72]. Most studies in this review, defined an adequate response as an IgG level above 1.00 µg/ml and/or two-fold increase in IgG level from baseline, which may be a good estimate for protection against certain serotypes (19A). However, for other serotypes it may be an under- or over-estimation.^11^

A major limitation of this review, which is inherent to the literature that it includes, is that immunogenicity was only assessed for a limited number of pneumococcal serotypes, mostly serotypes included in PCV. Not a single study assessed the serotype-specific response to all pneumococcal serotypes included in PPSV23. Currently, serotype 8 is the most common serotype causing invasive pneumococcal disease among adults (18–24%). This serotype is covered by PPSV23 but not by PCV13. Yet, this serotype has only been examined in one single study [73]. We highly recommend that future studies on pneumococcal vaccination should broaden the spectrum of their serological assays, to also include PPSV23/nonPCV13 serotypes. Due to serotype replacement induced by national immunisation programmes, the proportion of disease caused by PPSV23/nonPCV13 serotypes has expanded rapidly [73]. This highlights the importance of the inclusion of PPSV23 as part of the immunisation schedule for PLWH, while the promising 20-valent conjugate vaccine, including serotype 8, is still under investigation [74]. In the future, a single dose of the novel 20-valent conjugate vaccine may replace a combination of PCV/PPSV23, although its immunogenicity has yet to be determined in PLWH. There are several potential advantages of the novel PCV20 over the combined schedule. First, a one-dose strategy will likely increase vaccine uptake and adherence, especially in resource-poor settings with high prevalence of HIV where access to health care centres is challenging. In addition, a more durable immune response is expected by induction of immunological memory against a wider range of serotypes allowing for the possibility of longer time-intervals between booster doses. On the other hand, at lower CD4-counts PCV-20 may be less immunogenic compared to the current combination of PCV/PPSV23.

Moreover, a limitation of the meta-analysis was the high degree of heterogeneity that we observed for some subgroup analyses. Results of these analyses are therefore less precise and must be interpreted with caution. There are several possible explanations for the heterogeneity, including age differences between studies, differences in laboratory methods, different time-intervals between vaccinations and antibody assessments, and immunological differences between patient cohorts. Older age, lower CD4 counts, longer time-intervals between vaccination and lower cART coverage may negatively impact vaccination responses. We addressed this heterogeneity by using the random effects model estimates for all analyses, performing subgroup analyses based on CD4-cell count and cART status. The latter did not reduce heterogeneity. Meta-regression is another suitable method for exploring heterogeneity, however several authors could not provide the data needed for such an analysis. To reduce heterogeneity in future research on pneumococcal vaccines, it is recommended that standardized serological methods are used such as the WHO ELISA protocol, as well as uniform criteria for serologic response definition in adults [12,72].

Another limitation is that only 19/39 studies could be included in the meta-analysis. Not all authors responded to our data request, or did not have the necessary data available (Supplementary material Table 1 shows which authors responded to our data request). As a result, the number of studies and therefore patients included in the meta-analysis in which different vaccination strategies were compared was limited (159–440 individuals per analysis), and therefore a critical interpretation of the outcomes of these comparisons is required. It is important to note that a lack of statistical significance does not always infer a lack of clinical significance.

In conclusion, the evidence gathered here supports wide implementation of the combination of PCV/PPSV23 for all PLWH. We recommend reassessment of this strategy once higher-valent PCVs become available. The current review shows good overall immunogenicity of the combination of PCV/PPSV23 in the era of advanced cART. However, the durability of this vaccination schedule remains unknown and must be addressed in future research. Vaccination with PCV should be delayed until immunological recovery (CD4>200) in recently diagnosed PLWH for optimal vaccination responses. It is highly recommended that future studies on pneumococcal vaccines include a broader spectrum of serotypes, including PPSV23/non-PCV13 serotypes and use uniform serologic criteria in their analysis.

## Funding

H.M. Garcia Garrido is funded by a research grant of 10.13039/501100001826ZonMw (Project No. 522004005). No other funding was received for this study.

## Data Sharing Statement

Upon publication the datasets used for the meta-analysis will be freely available upon request. The corresponding author has access to all the datasets.

## Contributors

HMGG, AG and AV conceived the study. RS and HG designed the database search, RS performed the search. AG and HG screened and selected the studies. JLS and HMGG performed the data collection and risk of bias assessment. MWTT and HMGG performed the meta-analysis. HMGG and AG wrote the first version of the manuscript. MPG, AG, AV, JLS, RS, MWTT contributed to the data interpretation, reviewed the manuscript and contributed to the writing of the final manuscript. All authors approved of the final version of the manuscript.

## Declaration of Competing Interest

H.M. Garcia Garrido reports being funded by a public research grant from Zon MW, outside the submitted work. None of the other authors report a conflict of interest. All authors attest they meet the ICMJE criteria for authorship.

## Appendix Supplementary materials

Image, application 1

Image, application 2
