# Supplementary material for: Immunogenicity of pneumococcal vaccination in HIV infected individuals: A systematic review and meta-analysis
Source: eClinicalMedicine. 2020 Nov 23;29-30:100576. doi: 10.1016/j.eclinm.2020.100576 (PMC7695973; doi:10.1016/j.eclinm.2020.100576)
Supplement: Supplementary file 1 [file mmc1.pdf]

Supplementary File 1. Full search strategy

Embase Classic+Embase <1947 to 2020 June 10>		
Search history sorted by search number ascending		
#	Searches	Type
1	exp Human immunodeficiency virus/ or exp acquired immune deficiency syndrome/ or exp Human immunodeficiency virus infection/ or (PLWH or HIV* or "Human immunodeficiency virus" or AIDS or Acquired Immunodeficiency Syndrome or AIDS).ti,ab,kw.	Advanced
2	Pneumococcus vaccine/ or ("13-valent pneumococcal vaccine" or "23-valent pneumococcal polysaccharide vaccine" or Prevenar* or PCV13 or PCV7 or PCV10 or pneumovax* or PPSV23 or pneumococcal vaccin* or pneumococcal immune*).ti,ab,kw.	Advanced
3	1 and 2	Advanced
4	(rat or rats or mouse or mice or swine or porcine or murine or sheep or lambs or pigs or piglets or rabbit or rabbits or cat or cats or dog or dogs or cattle or bovine or monkey or monkeys or trout or marmoset$1).ti. or (Animal experiment/ not (human experiment/ or human/))	Advanced
5	3 not 4	Advanced
6	exp case report/	Advanced
7	5 not 6	Advanced
8	limit 7 to (conference abstracts or embase)	Advanced

Ovid MEDLINE(R) ALL <1946 to June 09, 2020>			
Search history sorted by search number ascending			
#	Searches		Type

1	exp "HIV"/ or exp "Acquired Immunodeficiency Syndrome"/ or (PLWH or HIV* or "Human immunodeficiency virus" or AIDS or Acquired Immunodeficiency Syndrome or AIDS).ti,ab,kf.		Advanced
2	exp "Pneumococcal Vaccines"/ or ("13-valent pneumococcal vaccine" or "23-valent pneumococcal polysaccharide vaccine" or Prevenar* or PCV13 or PCV7 or PCV10 or pneumovax* or PPSV23 or pneumococcal vaccin* or pneumococcal immune*).ti,ab,kf.		Advanced
3	1 and 2		Advanced
4	case reports.pt.		Advanced
5	3 not 4		Advanced
6	exp animals/ not humans/		Advanced
7	5 not 6	5	Advanced

Supplementary File 2. Explanation of Newcastle Ottawa risk of bias assessment tool for cohort studies

**Selection domain**	
Representativeness of exposed cohort (max: ✪)	Awarded if HIV+ patients were representative for current HIV+ population: approximately 70% males; average age >40 years old; >75% on antiretroviral therapy; mean CD4>400
Selection of non-exposed cohort (max: ✪)	Awarded if study had a control group of HIV-negative individuals or unvaccinated HIV+ control group.
Ascertainment of exposure (max: ✪)	Awarded if vaccinations were administered during the study or vaccine administration was registered in the patient file/registry.
Outcome was not present at start of study (max: ✪)	Awarded if baseline IgG/OPA assessment was done
**Comparability domain**	
Comparability of cohorts on the basis of the design or analysis (max ✪✪)	Awarded if comparative analyses were adjusted/matched for age (1 star) and at least one other important factor such as cART use or CD4 count (2 stars).
**Outcome domain**	
Assessment of outcome (max: ✪)	Awarded if serological methods were sufficiently described, primary outcomes were predefined and reported and, when applicable if cut-offs for seroconversion/seroprotection were predefined and supported by previous literature.
Follow-up long enough(max: ✪)	Awarded if post-vaccination antibody assessment was done at least 4 weeks after vaccination.
Adequacy of follow-up of cohorts (max: ✪)	Awarded if loss to follow up or missing data was <10%

Thresholds for converting the NOS rating to Agency for Healthcare Research and Quality - AHRQ - standards (good, fair, and poor):
Good quality: 3 or 4 stars in Selection domain AND 1 or 2 stars in Comparability domain AND 2 or 3 stars in Outcome domain
Fair quality: 2 stars in Selection domain AND 1 or 2 stars in Comparability domain AND 2 or 3 stars in Outcome domain
Poor quality: 0 or 1 star in Selection domain OR 0 stars in Comparability domain OR 0 or 1 stars in Outcome domain

Supplentary file 3 – Table 1: Additional characteristics of included studies.

Study characteristics									Patient characteristics			Outcomes				
**Study (year)** [*]	**Country**	**Design**	**Serologic methods**	**Correlate of protection**	**Sero-types assessed**	**Interval vaccination/ antibody assessment**[†]	**Previous pneumococcal vaccination**	**Vaccination schedule**	**Sub-jects at baseline (n)**	**Age (mean/median)**	**VL**[§] **un-decectable %**	**Seroprotection n/total (%)**	**Pre/Post-vaccination concentration of IgG (mcg/ml)** 6B	14	**Authors contacted for additional data**	**Response**
**Almeida (2009)** [23]	Brazil	Prospective cohort	ELISA[ǀ]	NR[¶]	1,3,5,6B, 9V,14	21-78 days (median 40)	0%	PPSV23 [#] (pregnant HIV[Δ]+ women)	44	26	NR	NR	1.18/3.05	3.20/9.39	No, data available from publication.	NA
**Amendola (2002)** [22]	Italy	Prospective cohort	ELISA	NR	3,4,6B, 19F, 23F	30 days	NR	**PPSV23** CD4 <200	10	37	NR	NR	1.9/4.0	NR	Yes	No
								CD4 200-500	23		NR	NR	2.6/8.5	NR		
								CD4 >500	24		NR	NR	3.6/4.7	NR		
								HIV- controls	20	NR	NR	NR	1.3/2.9	NR		
Belmonti (2019) [24]	Italy	Follow up Lombardi et al (2016); Prospective cohort	ELISA	IgG ≥1.0 mcg/ml **f**or ≥9 serotypes (70%)	1,3,4,5, 6A, 6B, 7F, 9V, 14, 18C, 19A, 19F, 23F	5 years	0%	PCV13[◊]+PCV13 (wk 0, 8)	50	44	92%	23/42 (55)	1.38 / 2.57	5.75 / 9.12	Yes	No
								PPSV23 (wk0)	50	46	84%	24/49 (49)	1.55 / 1.74	6.92 / 11.5		
Bhorat (2015) [25]	South Africa, Romania	Prospective cohort	ELISA + OPA[↓]	NR	1,3,4,5,6A,6B, 7F, 9V, 14, 18C, 19A, 19F, 23F	1,2,3,**4 months**	0%	3x PCV13 (mo 0,1,2) + PPSV23 (mo 3)	151 adults	41	71%	NR	3.13 /8.25	2.45 / 16.24	Yes	No
Chang (2000) [26]	United States	Prospective cohort	ELISA	NR	Total PPSV23 antipneumococcal IgG (ELISA)	28 days	0%	PPSV23 HIV+	12	41	50%	NR	*Inverse IgG titer 11.000 (from graph)*		Yes	Yes, but data not available.
								HIV- controls	10	35	NA	NR	*Inverse IgG titer 40.000 (from graph)*			
Cheng (2016) [46]	Taiwan	Prospective cohort (follow-up Lu 2012)	ELISA	IgG ≥1.0 mcg/ml	6B, 14, 19F, 23F	2,3,4 **5 years**	0%	PCV7 (wk0)	109	36	44%	6B: 85/102 (83); 19F: 101/102 (99); 23F: 69/102 (68)	0.855/ 1.79	1.992 / 6.98	No, data available from publication.	NA
								PCV7+PCV7 (wk0,4)	112	36	44%	6B: 82/103 (80) 19F: 97/103 (94) 23F: 76/103 (74)	0.774/ 1.73	1.91 / 8.22		

**Crum-Cianflone (2010)** [48]	United States	RCT☞	ELISA	≥1.0 mcg/ml	4, 9V, 14, 19F	14 days, **2**, 6 **months**	100% of HIV+	PPSV23 HIV+	73	42	64%	NR	NR	*1.0/2.0 (from graph)*	Yes	No
								PCV7 HIV+	131	42	70%	NR	NR	*1.2/3.3 (from graph)*		
							0% HIV-	PCV HIV-	25	37	NR	NR	NR	*0.1/4.8 (from graph)*		
**Deloria-Knoll (2006)** [50]	United States	RCT (Vitamin A, Zinc, both or Placebo)	ELISA	NR	4, 6B, 9V, 14, 18C, 19F, 23F	1,6,**7** months	0%	PCV 7 + PPSV23 (mo 0, mo 6)	118	42	13%	NR	1.47-3.21/ 2.96-7.09	NR	Yes	Yes
**Falco (2006) Vaccine** [27]	Spain	Prospective cohort	ELISA	NR	1, 6B, 14, 19F, 23F	**1,** 12 months	0%	PPSV23 (HIV+)	113	39	79%	NR	5.99 / 7.64	3.6 /12.1	Yes	Yes
								PPSV23 (HIV-)	30	34			2.95 / 5.45	1.82 / 7.55		
**Farmaki (2018)** [5]	Greece	Prospective cohort	ELISA	NR	3,14	1, **13 months**	70% between 1-5 years earlier	PCV13+PPSV23 (0,12 mo)	40	51	100%	NR	NR	1.21 / 4.27	Yes	Yes
**Feikin (2001)** [51]	United States	RCT	ELISA + OPA	NR	4, 6B, 9V, 14, 23F	8,9,**16,**17 ,24 weeks	0% in 5 years prior to study	PCV7+PCV7 (0,8wk)	15	45	NR	NR	0.99/ 2.0	5.0 / 10.3	Yes	No
								PCV7+PPSV23 (0,8wk)	18	42	NR	NR	1.2 / 2.2	5.0 / 11.4		
								Placebo+PPSV23 (0,8wk)	16	41	NR	NR	1.2 / 1.6	7.3 / 15.4		
								2 doses of placebo (0,8wk)	18	44	NR	NR	1.0/1.0	3.8/3.9		
Glesby (2015) [28]	United States	Prospective cohort	ELISA + OPA	NR	1,3,4,5, 6A, 6B, 7F, 9V, 14, 18C, 19A, 19F, 23F	1,7,**13** months	Yes 100% PPSV23	PCV13+PCV13+PCV13 (0,6,12 mo)	329	47	74%	NR	1.17 / 5.48	2.40 / 8.13	Yes	Yes, but data owned by Pfizer could not be provided unconditionally
Hart (2007) [29]	United Kingdom	Prospective cohort	ELISA	≥ 225 U/ml	Total PPSV23 anti-pneumococcal **IgG** and IgM	**1**, 3-6 months	NR	PPSV23 HIV+	33	46	100%	7/18 (39)	NR	NR	Yes	No
								HIV- controls	NR	31	NR	7/8 (88)	NR			
Ho (2013) [52]	Brazil	Randomised clinical trial	ELISA	≥1.00mcg /ml	6B, 9V, 14	2,**6** months		PPSV23+Placebo (mo 0, mo 2)	111	40	64%	6B: 47/89 (53) 14: 79/89 (89)	0.73/ 1.02	1.82 / 6.72	Yes	No

							0%	PCV7/Placebo (mo 0, mo 2)	110	40	67%	6B: 45/91 (50) 14: 76/89 (84)	0.51 / 1.24	1.15 / 9.32		
								PCV7/PPSV23 (mo 0, mo 2)	110	42	73%	6B: 54/91 (59) 14: 77/91 (85)	0.57 / 1.76	1.40 / 6.35		
Horster (2010) [30]	Germany	Prospective cohort	ELISA	NR	Total PPSV23 antipneumococcal IgG (ELISA)	1-13 months (median 4)	Not in 5 years prior to study	PPSV23	100	40	NR	NR	NR	NR	No, data available from publication.	NA
Huang (2018) [31]	China	Prospective cohort	OPA	NR	6B, 19A, 19F, 23F	1 month	0%	PPSV23 (HIV+)	63	38	NR	NR	27/901	NR	No (methods not suitable for meta-analysis)	NA
								PPSV23 (HIV-)	56	64	NA		85/2650	NR		
Hung (2010) [32]	Taiwan	Prospective cohort, follow-up report of Lu 2013	ELISA	≥0.35 mcg/ml	14, 19F, 23F	5 years	0%	PPSV23 CD4 <100	35	39	77%	*14: 30%; 23F: 10% (from graph)*	NR		Yes	Yes, however requested data not available.
								CD4 100-199	36	41	91%	*14: 60%; 23F: 30% (from graph)*	NR			
								CD4 200-349	34	42	94%	*14: 55%; 23F: 30% (from graph)*	NR			
								CD4 ≥350	64	39	81%	*14: 60%; 23F: 35%) (from graph)*	NR			
Kang (2016) [33]	United Kingdom	Retrospective cohort	NR	≥0.35 mcg/ml for ≥ 6 serotypes	NR	NR	NR	PPSV23	435	43	NR	258/435 (59)	NR		Yes	No
Leggat (2015) [34]	United States of America	Prospective cohort	ELISA + OPA	NR	14, 23F	1 month	0%	PPSV23 CD4>200 cART-	20	28	NR	NR	NR	5.2 / 27.8	Yes	Yes, but data could not be provided timely
								CD4<200 cART-	12	36	NR	NR	NR	6.9 / 26.0		

								CD4<200 cART+	11	45	NR	NR	NR	5.0/ 9.6		
								HIV- controls	22	26	NR	NR	NR	NR		
**Lesprit (2007)** [53]	France	RCT	ELISA	≥1.00mcg /ml	1,4,5 6B, 9V, 14, 18C, 19F, 23F	**8 weeks**, 24 weeks	Not in 5 years prior to study	PCV7+PPSV23 (wk 0, wk 4)	105	44	66%	NR	0.47/ 2.18	1.68 / 9.34	Yes	Yes
								PPSV23 (wk4)	103	45	58%	NR	0.47/ 1.55	1.07 / 5.88		
**Lombardi (2016)** [13]	Italy	Prospective cohort	ELISA	≥1.0 mcg/ml	1,3,4,5,6A, 6B, 7F, 9V, 14, 18C, 19A, 19F, 23F;	8,**24**,48 weeks	0%	PCV13+PCV13 (wk 0, 8)	50	44	84%	6B 44/46 (96); 14 45/46 (98) 23F 43/46 (93)	1.41 / 4.05	5.59 / 15.11	Yes	Yes
								PPSV23 (wk0)	50	46	92%	6B 44/49 (90);14 49/49 (100); 23F 44/49 (90)	1.53 / 2.69	6.69/18 .69		
**Lu (2012)** [36]	Taiwan	Prospective cohort	ELISA	≥1.00mcg /ml	6B, 14, 19F, 23F	**12**, 24, 36, 48 weeks	0%	PCV7 (wk 0)	114	38	46%	NR	NR	NR	Yes	Yes, however requested data not available.
								PCV7+PCV7 (wk 0 ,wk 4)	115	37	46%	NR	NR	NR		
Lu (2014) [35]	Taiwan	Prospective cohort (follow-up Hung 2010)	ELISA	≥1.00mcg /ml	6B, 14, 19F, 23F	12, 24, 36, **48 weeks**	Yes 100% received PPSV23 5 years earlier (Hung 2010)	PPSV23 (wk 0)	127	45	83%	NR	NR	NR	Yes	Yes, however requested data not available.
								PCV7 (wk 0)	50	44	82%	NR	NR	NR		
								PCV7+PCV7 (wk 0 ,wk 4)	44	43	84%	NR	NR	NR		
Lu (2013) [37]	Taiwan	Prospective cohort (PPSV23 arm = same cohort as Hung 2010; PCV7 arm = same cohort as Lu 2012)	ELISA	NR	6B, 14, 19F, 23F	24, **48 weeks**	0%	PPSV23	169	39	84%	NR	NA	2.35/ 3.14	Yes	Yes, however requested data not available.
								PCV7	114	39	59%	NR	0.76 / 1.06	1.83/5. 00		
MacLennan (2016) [38]	United Kingdom	Prospective cohort	Multiplex fluorescence	≥1.3 mcg/ml for 8/12 serotypes (67%)	1, 3, 4, 5, 6B, 7F, 9V, 14, 18C, 19A, 19F, and 23F	4-6 weeks	0%	PPSV23 (HIV+)	211	35	73%	6B: 44% | 14: 75% | 23F: 43% Overall: 33%	0.32 / 0.97	1.51 / 5.12	Yes	No

			microbead assay					PPSV23 (HIV-)	73	40		6B: 55% | 14: 74% | 23F: 63% Overall: 53%	0.36 / 1.67	1.03 / 7.76		
**Ohtola (2016)** [39]	United States	Prospective cohort	ELISA + OPA	≥1.00mcg /ml	PCV13/PPSV23: 14, 23F	1 month after PPSV23	87% HIV+; 7% HIV- >5 years prior to study	PPSV23 (HIV+)	22	55	100%	NR	NR	NR (only in graph)	Yes	Yes, but data could not be provided timely
								PCV13+PPSV23 (wk 0, 8 HIV+)	15	55	100%	NR		NR (only in graph)		
								PCV13+PPSV23 (wk 0, 8 HIV-)	14	57	NA	NR		NR (only in graph)		
Payeras (2002) [40]	Spain	Prospective cohort	ELISA + OPA	NR	Total PPSV23 antipneumococcal IgG (ELISA); OPA for serotype 3	4 weeks	0%	PPSV23 (HIV+ and recurrent bacterial infection)	33	35	NR	NR	874 U/ml		Yes	No
								PPSV23 (HIV+ controls)	33	37	NR	NR	1265 U/ml			
								PPSV23 (HIV-)	NR	NR	NA	NR	2501 U/ml			
**Peñaranda (2010)** [54]	Spain	RCT	ELISA	≥1.00mcg /ml	4, 6B, 9V, 14, 18C, 19F, 23F;	4, **8** weeks	0%	PCV7/PPSV23 (wk0, wk4)	110	44	100%	NR	NR	NR	Yes	No
								PPSV23 (wk 0)	110	44	100%	NR	NR	NR		
**Rash (2015)** [41]	Canada	Prospective cohort	NR	≥1.3 mcg/ml	NR	4 weeks	NR	PPSV23	23	NR	100%	NR	NR	NR	Yes	No
Rodriguez-Barradas (2003) [42]	United States	Prospective cohort	ELISA			4-12 weeks	n=41 (analysed seperately)	PPSV23 (cART+), first time	46	47	46%	NR	0.76 / 1.40	NR	Yes	Yes, but data could not be provided timely
								PPSV23 (cART+) second time	41	47	46%	NR	0.96 / 1.95	NR		
								PPSV23 (cART-)	38	42	0	NR	0.88 / 3.12	NR		
**Rodriguez-Barradas (2015)** [49]	United States	RCT (immediate versus delayed PPSV23 vaccination)	**ELISA** + OPA	≥1.0 mcg/ml	1,3,4, 6B, 23F	**1**,6,12 months after each intervention	PPSV23 > 3 years earlier 20%	PPSV23/Placebo (mo 0, mo 9-12)	36	44	0%	NR	5.12 / 7.85	NR	Yes	Yes, but data could not be provided timely
								Placebo/PPSV23 (mo 0, mo 9-12)	36	45	60%	NR	5.17 / 5.79			
Rossheim (2016) [43]	United States	Prospective cohort	ELISA	NR	3, 6A, 7F, 19A	**1, 12 months**	100% PPSV23 prior to study	PCV13 (PPSV23 1-3 years earlier)	42	46	100%	NR	NR	NR	Yes	Yes, but data could not be provided.
								PCV13 (PPSV23 >3 years earlier)	54	42	100%	NR	NR	NR		
**Sadlier (2016)** [55]	Ireland	RCT	**ELISA** + OPA	≥1.0 mcg/ml	1,3,4,5, 6B, 7F, 9V, 14,	**8,** 28 weeks	0%	PCV13+PPSV23 (wk 0, 4)	28	37	NR	NR	0.18/ 1.56	0.44 / 4.61	Yes	No

					18C, 19A, 19F, 23F			PPSV23 (wk 4)	33	36	NR	NR	0.18 / 0.81	1.04 /5.71		
**Sögaard (2010)** [47]	Denmark	RCT	**ELISA** + OPA	≥1.0 mcg/ml	**PCV7/PPSV23:** 4,6B, 9V, 14, 18C, 19F, 23F; **PPSV23**: 1, 7F, 19A	3,4,9,**10 months**	n=3 PPSV23 (>5 years earlier)	PCV7/PCV7/PPSV23 (mo 0, 3,9)+placebo	49	49	100%	NR	1.23 / 4.07	2.38 /10.1	Yes	Yes
								PCV7/PCV7/PPSV23 (mo 0, 3,9) + CPG7909 adjuvans	48	49	100%	NR	1.03 / 5.21	1.92 / 9.76		
**Slayter (2013)** [14]	Canada	RCT Immediate versus delayed immunization	ELISA + OPA	NR	**PCV7/PPSV23:** 4, 6B, 9V, 14, 18C, 19F, 23F	**1**,6,12 months	0%	PCV7 (immediate/delayed)	(23 | 16)	(43|42)	NR	NR	0.20/ 0.55	0.25 / 0.92	Yes	Yes
								PPSV23 (immediate/delayed)	(19 | 21)	(40|38)	NR	NR	0.14 / 0.43	0.25 /0.80		
Song (2019) [44]	Korea	Prospective cohort	Multiplex OPA	OPA titer > ≥1:64	5,6B,18C, 19A	21-35 days	0%	PCV13 (CD4 >350)	34	41	62%	34/34 (100)	NR	NR	No (methods not suitable for meta-analysis)	NA
								PCV13 (CD4<350)	33	42	52%	29/33 (88)	NR	NR		
**Tasker (2002)** [57]	United States	RCT / Prospective cohort study (combined)	ELISA	NR	4, 6B, 9V, 14	**6**,12 weeks	n= 85 (>5 years prior to study)	PPSV23 first time (cART-)	14	27	0%	NR	NR	NR	Yes	Yes, however data not available
								PPSV23 second time (cART+)	56	26	NR		NR	NR		
								Placebo (second time cART+)	29	37	NR		NR	NR		
**Tsachouridou (2015)** [45]	Greece	Prospective cohort	ELISA	NR	Total PPSV23 antipneumococcal IgG	**4**, 48 weeks	0%	PPSV23 (cART+)	35	33	NR	NR	NR	NR	Yes	No
								PPSV23 (cART-)	31	30	NR		NR	NR		
NCT02717494 (2020) [56]	Brazil	RCT (pregnant HIV+ women)	ELISA	≥0.35 mcg/ml	NR	4 weeks	0%	PCV10	115	27	NR	114/114 (100)	NR	NR	Yes	Yes, but data could not be provided.
								PPSV23	115	28		109/110 (99)	NR	NR		
								Placebo	116	28		106/113 (94)	NR	NR		

Legend supplementary table 1

* Studies printed in bold were included in the meta-analysis

† If multiple time-points were assessed, results are shown for the bold printed time-point
‡ cART= combination antiretroviral treatment, defined as a combination of at least antiretroviral drugs
§ VL= viral load, undetectable viral load was defined as a value below the lowest level of detection in individual studies
| ELISA= Enzyme-Linked Immuno Sorbent assay
¶ NR = not reported
# PPSV23 = 23-valent pneumococcal polysaccharide vaccine
Δ HIV= human Immunodeficiency virus
◊ PCV= pneumococcal conjugate vaccine
↓ OPA= opsonophagocytic assay
☞ RCT = Randomized clinical trial

Supplementary figure 1: Forest plot of studies comparing seroconversion rates for the pneumococcal conjugated vaccine (PCV) versus the pneumococcal polysaccharide vaccine (PPSV) – fixed effects model (corresponding to figure 5)

A Serotype 6 B

	PCV		PPSV23			Odds Ratio
Study or Subgroup	Events	Total	Events	Total	Weight	M-H, Fixed, 95% CI
Lombardi 2016	27	46	20	49	50.6%	2.06 [0.91, 4.67]
Slayter 2013 (delayed)	7	10	6	17	8.4%	4.28 [0.80, 22.93]
Slayter 2013 (immediate)	7	19	10	18	41.0%	0.47 [0.13, 1.74]
**Total (95% CI)**		**75**		**84**	**100.0%**	**1.59 [0.86, 2.97]**
Total events	41		36			

Heterogeneity: Chi² = 5.05, df = 2 (P = 0.08); I² = 60%
Test for overall effect: Z = 1.47 (P = 0.14)

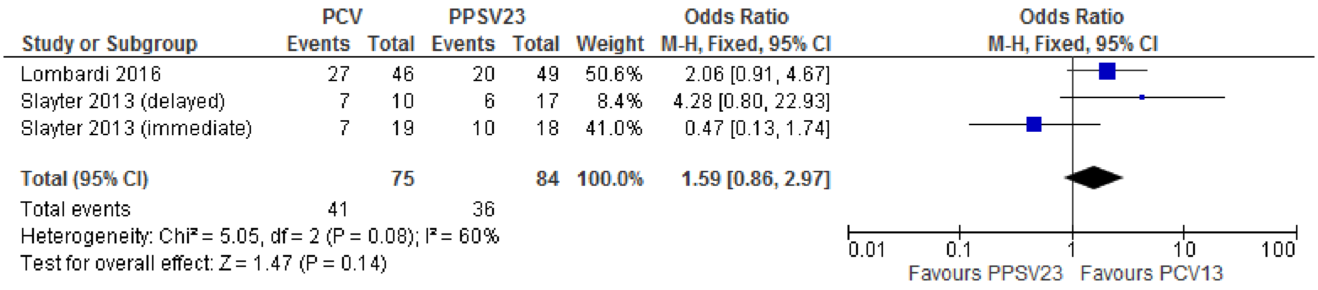

B Serotype 14

	PCV		PPSV23			Odds Ratio
Study or Subgroup	Events	Total	Events	Total	Weight	M-H, Fixed, 95% CI
Crum-Cianflone 2010	61	120	28	64	48.8%	1.33 [0.72, 2.45]
Lombardi 2016	28	46	33	49	34.0%	0.75 [0.33, 1.75]
Slayter 2013 (delayed)	7	10	11	17	6.6%	1.27 [0.24, 6.82]
Slayter 2013 (immediate)	10	19	8	18	10.6%	1.39 [0.38, 5.07]
**Total (95% CI)**		**195**		**148**	**100.0%**	**1.14 [0.73, 1.77]**
Total events	106		80			

Heterogeneity: Chi² = 1.28, df = 3 (P = 0.73); I² = 0%
Test for overall effect: Z = 0.57 (P = 0.57)

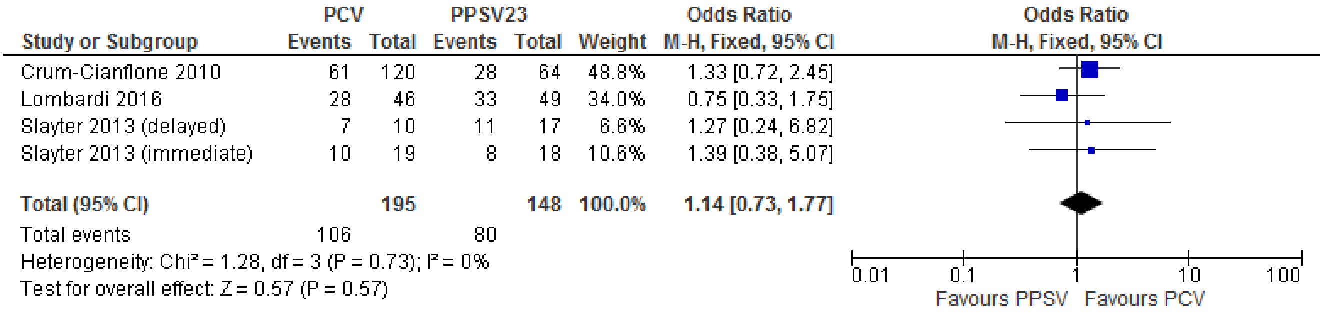

C Overall

	PCV		PPSV			Odds Ratio
Study or Subgroup	Events	Total	Events	Total	Weight	M-H, Fixed, 95% CI
Crum-Cianflone 2010	68	120	23	64	39.2%	2.33 [1.25, 4.36]
Lombardi 2016	10	46	14	49	32.0%	0.69 [0.27, 1.77]
Slayter 2013 (delayed)	7	10	11	17	7.4%	1.27 [0.24, 6.82]
Slayter 2013 (immediate)	7	19	11	18	21.5%	0.37 [0.10, 1.40]
**Total (95% CI)**		**195**		**148**	**100.0%**	**1.31 [0.83, 2.05]**
Total events	92		59			

Heterogeneity: Chi² = 8.49, df = 3 (P = 0.04); I² = 65%
Test for overall effect: Z = 1.17 (P = 0.24)

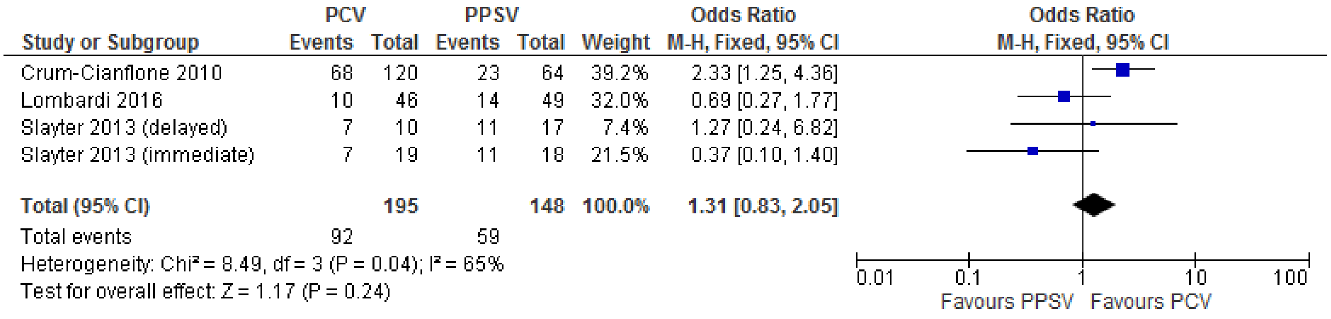

Supplementary figure 2: Forest plot of studies comparing seroconversion rates for the combined pneumococcal vaccination schedule (PCV+PPSV) versus the pneumococcal polysaccharide vaccine (PPSV) alone – fixed effects model (corresponding to figure 6)

A Serotype 6B

Study or Subgroup	PCV+PPSV Events	PCV+PPSV Total	PPSV Events	PPSV Total	Weight	Odds Ratio M-H, Fixed, 95% CI
Feikin 2001	8	18	3	16	3.8%	3.47 [0.73, 16.53]
Lesprit 2007	57	105	53	103	52.6%	1.12 [0.65, 1.93]
Peñaranda 2010	31	98	30	100	43.6%	1.08 [0.59, 1.97]
**Total (95% CI)**		**221**		**219**	**100.0%**	**1.19 [0.81, 1.76]**
Total events	96		86			

Heterogeneity: Chi² = 1.95, df = 2 (P = 0.38); I² = 0%
Test for overall effect: Z = 0.88 (P = 0.38)

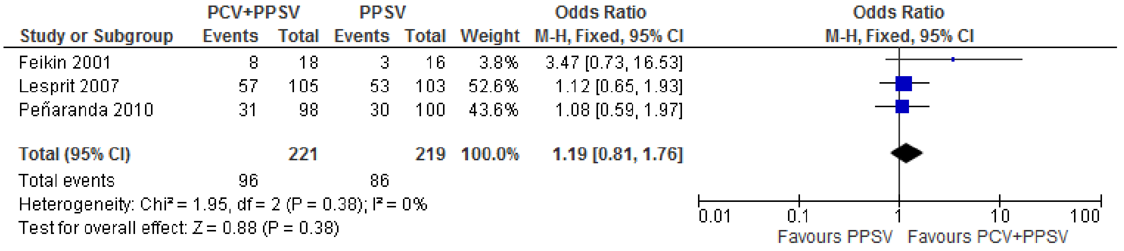

B Serotype 14

Study or Subgroup	PCV+PPSV Events	PCV+PPSV Total	PPSV Events	PPSV Total	Weight	Odds Ratio M-H, Fixed, 95% CI
Feikin 2001	7	18	7	16	7.4%	0.82 [0.21, 3.22]
Lesprit 2007	57	105	70	103	52.9%	0.56 [0.32, 0.98]
Peñaranda 2010	49	98	49	100	39.7%	1.04 [0.60, 1.82]
**Total (95% CI)**		**221**		**219**	**100.0%**	**0.77 [0.53, 1.12]**
Total events	113		126			

Heterogeneity: Chi² = 2.36, df = 2 (P = 0.31); I² = 15%
Test for overall effect: Z = 1.35 (P = 0.18)

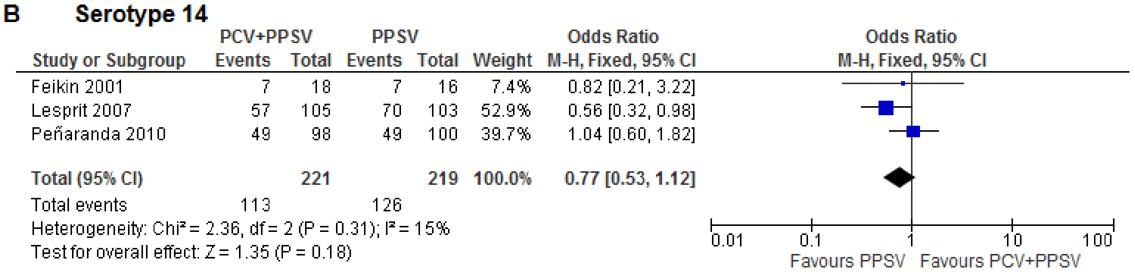

C Overall

Study or Subgroup	PCV+PPSV Events	PCV+PPSV Total	PPSV23 Events	PPSV23 Total	Weight	Odds Ratio M-H, Fixed, 95% CI
Lesprit 2007	62	105	41	103	71.8%	2.18 [1.25, 3.79]
Ohtola 2016	11	15	12	22	11.0%	2.29 [0.55, 9.47]
Sadlier 2016	16	26	11	28	17.2%	2.47 [0.83, 7.39]
**Total (95% CI)**		**146**		**153**	**100.0%**	**2.24 [1.41, 3.58]**
Total events	89		64			

Heterogeneity: Chi² = 0.04, df = 2 (P = 0.98); I² = 0%
Test for overall effect: Z = 3.39 (P = 0.0007)

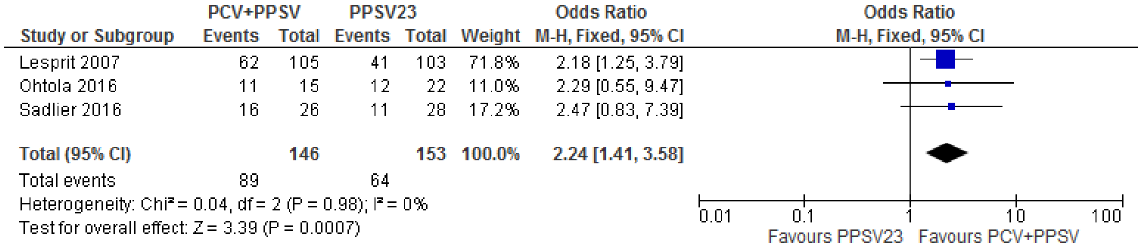

Supplementary figure 3: Forest plot of studies showing comparison of log-transformed geometric mean concentrations of IgG of serotype 6B (A and B) and serotype 14 (B and C) for the pneumococcal conjugated vaccine (PCV) versus the pneumococcal polysaccharide vaccine (PPSV)

Serotype 6B Baseline

	PCV+PPSV			PPSV				Mean Difference
Study or Subgroup	Mean	SD	Total	Mean	SD	Total	Weight	IV, Random, 95% CI
Feikin 2001	0.182	1.05	18	0.182	0.99	16	27.7%	0.00 [-0.69, 0.69]
Lesprit 2007	-0.76	1.31	105	-0.76	1.29	103	60.2%	0.00 [-0.35, 0.35]
Sadlier 2016	-0.71	2.76	27	-1.71	1.32	33	12.2%	1.00 [-0.13, 2.13]
**Total (95% CI)**			**150**			**152**	**100.0%**	**0.12 [-0.30, 0.54]**

Heterogeneity: Tau² = 0.04; Chi² = 2.77, df = 2 (P = 0.25); I² = 28%
Test for overall effect: Z = 0.57 (P = 0.57)

Mean Difference
IV, Random, 95% CI

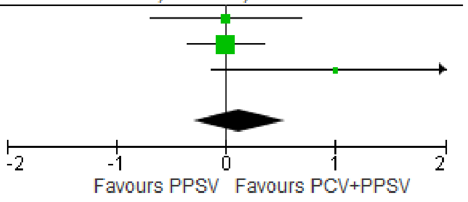

Serotype 6B Post-vaccination

	PCV			PPSV				Mean Difference
Study or Subgroup	Mean	SD	Total	Mean	SD	Total	Weight	IV, Random, 95% CI
Feikin 2001	0.693	1.25	18	0.47	1.35	16	9.9%	0.22 [-0.66, 1.10]
Ho 2013	0.631	1.71	100	0.372	1.68	100	34.7%	0.26 [-0.21, 0.73]
Lombardi 2016	1.4	1.04	46	1.21	0.967	49	46.8%	0.19 [-0.21, 0.59]
Slayter 2013	-0.59	1.83	29	-0.84	2.01	35	8.6%	0.25 [-0.69, 1.19]
**Total (95% CI)**			**193**			**200**	**100.0%**	**0.22 [-0.05, 0.50]**

Heterogeneity: Tau² = 0.00; Chi² = 0.05, df = 3 (P = 1.00); I² = 0%
Test for overall effect: Z = 1.58 (P = 0.12)

Mean Difference
IV, Random, 95% CI

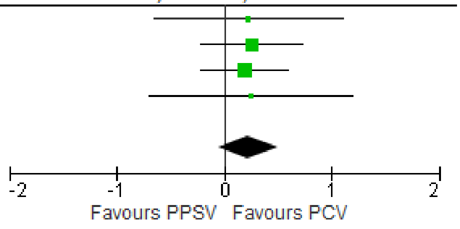

Serotype 14 Baseline

	PCV			PPSV23				Mean Difference
Study or Subgroup	Mean	SD	Total	Mean	SD	Total	Weight	IV, Random, 95% CI
Feikin 2001	1.61	1.13	18	1.99	0.944	16	19.7%	-0.38 [-1.08, 0.32]
Ho 2013	0.14	1.85	109	0.599	1.55	111	30.8%	-0.46 [-0.91, -0.01]
Lombardi 2016	1.72	0.995	50	1.901	0.974	50	34.6%	-0.18 [-0.57, 0.20]
Slayter 2013	-1.39	1.67	31	-0.04	1.97	38	14.9%	-1.35 [-2.21, -0.49]
**Total (95% CI)**			**208**			**215**	**100.0%**	**-0.48 [-0.87, -0.09]**

Heterogeneity: Tau² = 0.08; Chi² = 6.00, df = 3 (P = 0.11); I² = 50%
Test for overall effect: Z = 2.39 (P = 0.02)

Mean Difference
IV, Random, 95% CI

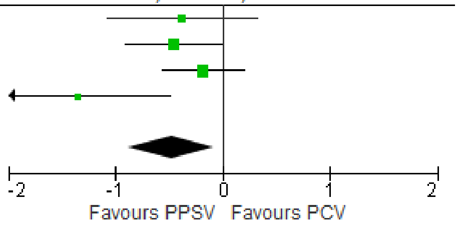

Serotype 14 Post-Vaccination

	PCV			PPSV23				Mean Difference
Study or Subgroup	Mean	SD	Total	Mean	SD	Total	Weight	IV, Random, 95% CI
Feikin 2001	2.37	1.11	18	2.73	1.14	16	19.9%	-0.36 [-1.12, 0.40]
Ho 2013	2.51	2.12	100	2.18	1.74	100	32.3%	0.33 [-0.21, 0.87]
Lombardi 2016	2.85	1.21	46	3.23	1.21	49	36.5%	-0.38 [-0.87, 0.11]
Slayter 2013	1.15	2.1	29	1.23	2.25	35	11.3%	-0.08 [-1.15, 0.99]
**Total (95% CI)**			**193**			**200**	**100.0%**	**-0.11 [-0.50, 0.27]**

Heterogeneity: Tau² = 0.04; Chi² = 4.18, df = 3 (P = 0.24); I² = 28%
Test for overall effect: Z = 0.57 (P = 0.57)

Mean Difference
IV, Random, 95% CI

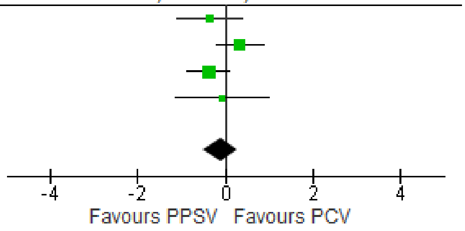

Supplementary figure 4: Forest plot showing comparison of log-transformed geometric mean concentrations of IgG of serotype 6B (A and B) and serotype 14 (B and C) for the combined vaccination schedule (PCV+PPSV) versus the pneumococcal polysaccharide vaccine (PPSV) alone.

A 6B Baseline

	PCV+PPSV			PPSV				Mean Difference
Study or Subgroup	Mean	SD	Total	Mean	SD	Total	Weight	IV, Random, 95% CI
Feikin 2001	0.182	1.05	18	0.182	0.99	16	27.7%	0.00 [-0.69, 0.69]
Lesprit 2007	-0.76	1.31	105	-0.76	1.29	103	60.2%	0.00 [-0.35, 0.35]
Sadlier 2016	-0.71	2.76	27	-1.71	1.32	33	12.2%	1.00 [-0.13, 2.13]
**Total (95% CI)**			**150**			**152**	**100.0%**	**0.12 [-0.30, 0.54]**

Heterogeneity: Tau² = 0.04; Chi² = 2.77, df = 2 (P = 0.25); I² = 28%
Test for overall effect: Z = 0.57 (P = 0.57)

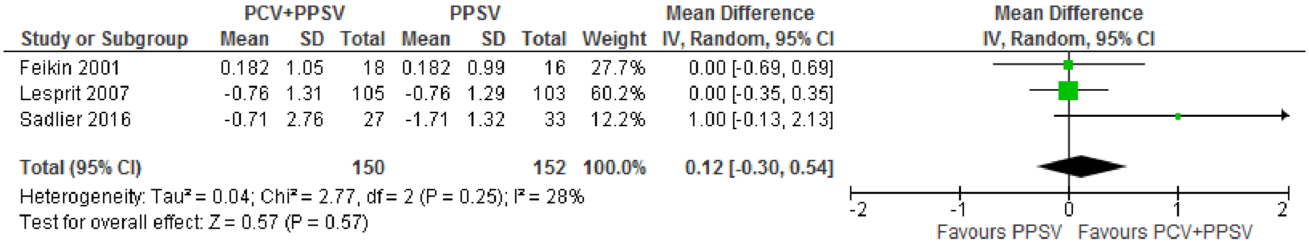

B 6B Post-vaccination

	PCV+PPSV			PPSV				Mean Difference
Study or Subgroup	Mean	SD	Total	Mean	SD	Total	Weight	IV, Random, 95% CI
Feikin 2001	0.788	1.13	18	0.47	1.35	16	26.2%	0.32 [-0.52, 1.16]
Lesprit 2007	0.445	1.92	105	0.438	1.38	103	56.4%	0.01 [-0.45, 0.46]
Sadlier 2016	0.779	1.61	26	-0.211	2.42	28	17.4%	0.99 [-0.10, 2.08]
**Total (95% CI)**			**149**			**147**	**100.0%**	**0.26 [-0.24, 0.76]**

Heterogeneity: Tau² = 0.06; Chi² = 2.79, df = 2 (P = 0.25); I² = 28%
Test for overall effect: Z = 1.02 (P = 0.31)

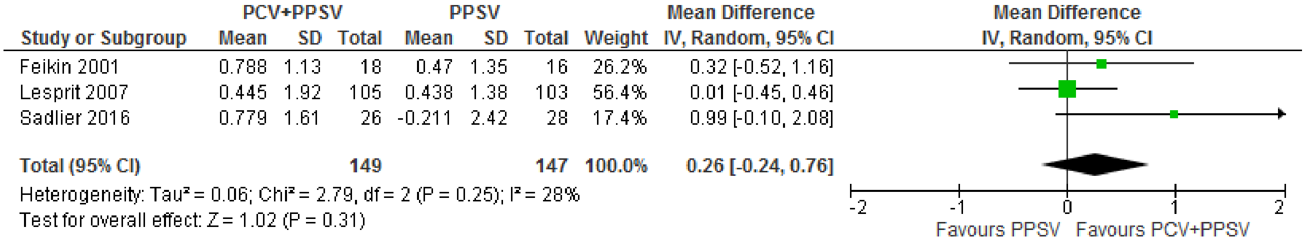

C 14 Baseline

	PCV+PPSV			PPSV				Mean Difference
Study or Subgroup	Mean	SD	Total	Mean	SD	Total	Weight	IV, Random, 95% CI
Feikin 2001	1.61	1.13	18	1.99	0.944	16	32.5%	-0.38 [-1.08, 0.32]
Lesprit 2007	0.519	1.45	105	0.068	1.76	103	38.2%	0.45 [0.01, 0.89]
Sadlier 2016	-0.82	1.82	27	0.039	1.42	33	29.3%	-0.86 [-1.70, -0.02]
**Total (95% CI)**			**150**			**152**	**100.0%**	**-0.20 [-1.00, 0.60]**

Heterogeneity: Tau² = 0.39; Chi² = 9.16, df = 2 (P = 0.01); I² = 78%
Test for overall effect: Z = 0.50 (P = 0.62)

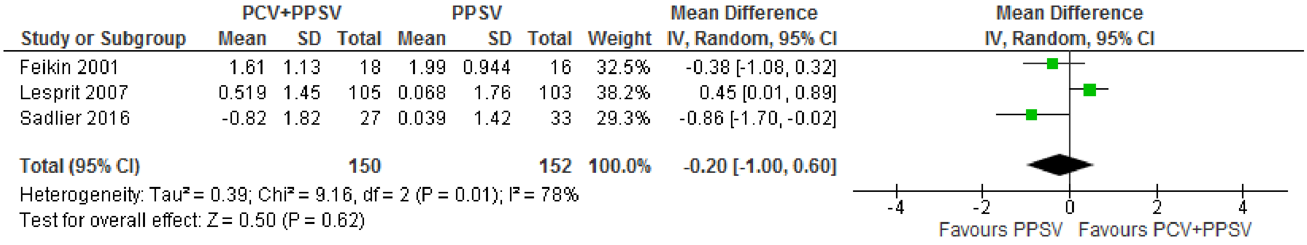

D 14 Post- Vaccination

	PCV+PPSV			PPSV				Mean Difference
Study or Subgroup	Mean	SD	Total	Mean	SD	Total	Weight	IV, Random, 95% CI
Feikin 2001	2.43	1.15	18	2.18	0.696	16	30.3%	0.25 [-0.38, 0.88]
Lesprit 2007	2.23	1.61	105	1.77	1.61	103	63.0%	0.46 [0.02, 0.90]
Sadlier 2016	1.53	3.14	26	1.74	1.61	28	6.7%	-0.21 [-1.56, 1.14]
**Total (95% CI)**			**149**			**147**	**100.0%**	**0.35 [0.00, 0.70]**

Heterogeneity: Tau² = 0.00; Chi² = 1.00, df = 2 (P = 0.61); I² = 0%
Test for overall effect: Z = 1.98 (P = 0.05)

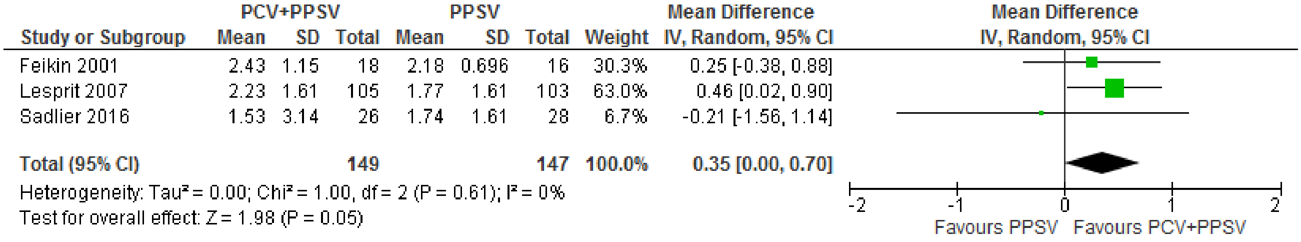

Box 1: Research questions

The proposed systematic review sought to answer the following research questions:

- What is the immunologic response to pneumococcal vaccines (polysaccharide, conjugate or both) in adult PLWH, either expressed in seroconversion rate (SCR), or geometric mean concentration (GMC) of anti-pneumococcal IgG levels, or opsonophagocytic activity (OPA)?
- Does the response to vaccination differ between different pneumococcal vaccination schedules?
- Does the response to vaccination differ between subgroups of PLHW, based on CD4 count, viral load and/or cART use?
- Is the immunologic response to vaccination durable in PLWH?

Box 2: Eligibility criteria

The following studies were included in the systematic review:

o Randomized controlled trials (RCT), prospective, and retrospective cohort studies, in which at least one dose of pneumococcal vaccine (either conjugate, or polysaccharide or both) was administered to adult PLWH.

o Studies performed in the era of advanced antiretroviral therapy, which is after the year 2000, when combination antiretroviral therapy became universally recommended.

o Studies reporting vaccine immunogenicity outcomes, including seroconversion rate (SCR), geometric mean anti-pneumococcal IgG levels, or opsonophagocytic activity.

We excluded paediatric studies; animal studies; case reports; studies that did not reported serological outcomes, and vaccination studies published before 2000, because pre- or early cART eras do not adequately reflect the current situation for PLWH.

The following studies were also included in the meta-analysis:

o Studies reporting seroconversion rates (SCR) for serotype 6B, 14, and/or the overall seroconversion rate, 1-3 months after vaccination, following one or more of the following definitions:

1. The n/total (%) of patients reaching a ≥ 2-fold increase in IgG for serotype 6B, or the n/total (%) of patients reaching a ≥ 2-fold increase AND IgG level ≥1.00 μg/ml for serotype 6B.
2. The n/total (%) of patients reaching a ≥ 2-fold increase in IgG for serotypes 14, or the n/total (%) of patients reaching a ≥ 2-fold increase AND IgG level ≥1.00 μg/ml for serotype 14.
3. The n/total (%) of patients reaching a ≥ 2-fold increase for at least 50-70% of measured serotypes, or the n/total (%) of patients reaching a ≥ 2-fold increase AND IgG level ≥1.00 μg/ml for at least 50-70% of measured serotypes (overall SCR).

o Studies comparing PPSV23 to PCV, or to the combination of PCV/PPSV23, and which reported baseline and post-vaccination GMCs with 95% confidence intervals 1-3 months after vaccination, for serotype 6B and/or 14. Studies from which the GMCs had to be extracted from graphs were excluded from meta-analysis.

<u>Box 3: Methods for meta-analysis</u>

We obtained pooled SCRs for serotypes 6B, 14, and the overall SCR, and associated 95% confidence intervals (CI) in PLWH, after single PCV, multiple PCVs, PPSV23, and PCV/PPSV23 combined, using R version 3.5.0. package ‘meta’, command ‘metaprop’. We used the I^2 test to indicate the level of statistical heterogeneity between studies. Odds ratios for SCR-differences between vaccination regimens were calculated in RevMan version 5.3. GMCs for serotype 6B and 14, and their 95% confidence intervals, were log transformed, and the standard error was calculated from the log-transformed 95% confidence interval. Log transformed data were entered in RevMan 5.3, using the inverse variance method, to calculate the GMC differences between vaccination schedules (PCV versus PPSV23, and PCV/PPSV23 combined versus PPSV23).

In view of the sample size and considerable statistical heterogeneity we used the random-effects model for all meta-analyses. We depicted the results of our meta-analysis in Forest plots. Where appropriate, we used the 95% confidence interval (CI) to indicate statistical significance. In addition, prediction intervals were calculated for all Odds ratios from the comparative meta-analysis, because a CI

provides an incomplete summary of the underlying heterogeneity in meta-analysis, whereas prediction intervals show the range of true effects as can be expected from future studies. Last, subgroup analyses were performed to investigate the influence of study quality, cART use, and CD4-cell counts on effect estimates and heterogeneity.
